# Using molecular similarity to highlight the challenges of routine immunoassay-based drug of abuse/toxicology screening in emergency medicine

**DOI:** 10.1186/1471-227X-9-5

**Published:** 2009-04-28

**Authors:** Matthew D Krasowski, Anthony F Pizon, Mohamed G Siam, Spiros Giannoutsos, Manisha Iyer, Sean Ekins

**Affiliations:** 1 Department of Pathology, University of Pittsburgh, Pittsburgh, PA, USA; 2 Department of Pathology, Division of Clinical Chemistry, Toxicology and Therapeutic Drug Monitoring Laboratory, University of Pittsburgh Medical Center Presbyterian/Shadyside, Pittsburgh, PA, USA; 3 Department of Emergency Medicine, Division of Medical Toxicology, University of Pittsburgh Medical Center, Pittsburgh, PA, USA; 4 Department of Forensic Medicine and Toxicology, Zagazig University, Zagazig, Egypt; 5 Collaborations in Chemistry, Jenkintown, PA, USA; 6 Department of Pharmacology, University of Medicine and Dentistry of New Jersey, Robert Wood Johnson Medical School, Piscataway, NJ, USA; 7 Department of Pharmaceutical Sciences, University of Maryland, Baltimore, MD, USA; 8 Department of Pathology (6233 RCP), The University of Iowa Hospitals and Clinics, 200 Hawkins Drive, Iowa City, IA 52242, USA

## Abstract

**Background:**

Laboratory tests for routine drug of abuse and toxicology (DOA/Tox) screening, often used in emergency medicine, generally utilize antibody-based tests (immunoassays) to detect classes of drugs such as amphetamines, barbiturates, benzodiazepines, opiates, and tricyclic antidepressants, or individual drugs such as cocaine, methadone, and phencyclidine. A key factor in assay sensitivity and specificity is the drugs or drug metabolites that were used as antigenic targets to generate the assay antibodies. All DOA/Tox screening immunoassays can be limited by false positives caused by cross-reactivity from structurally related compounds. For immunoassays targeted at a particular class of drugs, there can also be false negatives if there is failure to detect some drugs or their metabolites within that class.

**Methods:**

Molecular similarity analysis, a computational method commonly used in drug discovery, was used to calculate structural similarity of a wide range of clinically relevant compounds (prescription and over-the-counter medications, illicit drugs, and clinically significant metabolites) to the target ('antigenic') molecules of DOA/Tox screening tests. These results were compared with cross-reactivity data in the package inserts of immunoassays marketed for clinical testing. The causes for false positives for phencyclidine and tricyclic antidepressant screening immunoassays were investigated at the authors' medical center using gas chromatography/mass spectrometry as a confirmatory method.

**Results:**

The results illustrate three major challenges for routine DOA/Tox screening immunoassays used in emergency medicine. First, for some classes of drugs, the structural diversity of common drugs within each class has been increasing, thereby making it difficult for a single assay to detect all compounds without compromising specificity. Second, for some screening assays, common 'out-of-class' drugs may be structurally similar to the target compound so that they account for a high frequency of false positives. Illustrating this point, at the authors' medical center, the majority of positive screening results for phencyclidine and tricyclic antidepressants assays were explained by out-of-class drugs. Third, different manufacturers have adopted varying approaches to marketed immunoassays, leading to substantial inter-assay variability.

**Conclusion:**

The expanding structural diversity of drugs presents a difficult challenge for routine DOA/Tox screening that limit the clinical utility of these tests in the emergency medicine setting.

## Background

Medical complications related to drugs account for a significant fraction of patient visits to the emergency department (ED). These visits may be a result of illicit drug abuse, intentional or inadvertent overdose of prescription or over-the-counter medications, or drug-drug interactions [1-3]. There is increasing concern about the danger posed by misuse of prescription medications, particularly those with high potential abuse liability (e.g., opioids), especially when used in combination with ethanol or street drugs [4]. In some patients, such as those with altered mental status, a medical history may be unclear at the time of presentation to the ED. To aid in the diagnosis and management of drug-related complications, laboratory tests to screen for the presence of drugs and drug metabolites are widely used in emergency medicine [3,5]. We will refer to these tests as 'drug of abuse/toxicology (DOA/Tox) screening tests'.

Over the last four decades, a number of methods have been used for DOA/Tox screening including antibody-based assays (immunoassays) [6,7]. DOA/Tox immunoassay screens for amphetamines, barbiturates, benzodiazepines, cannabinoids, methadone, opiates, and tricyclic antidepressants (TCAs) were first introduced into clinical practice in the United States in the 1970s, initially as radioimmunoassays and later as non-radioactive immunoassays [8,9]. Immunoassays have steadily displaced other DOA/Tox screening methods such as thin-layer chromatography or colorimetric assays [7]. Currently, the most common methods used in the United States for DOA/Tox screening are homogeneous immunoassays that can be performed rapidly on a variety of different instruments, ranging from small devices that can be located within or near the ED to large, high-throughput analyzers found in hospital clinical laboratories or off-site reference laboratories [6,7]. Screening assays are different from confirmatory tests such as gas chromatography/mass spectrometry (GC/MS) that can provide definitive identification of individual drugs and their metabolites [7]. Confirmatory tests are often more labor-intensive, technically demanding, and expensive compared with screening tests. For many EDs, confirmatory tests are available only by referral of patient samples to an off-site reference laboratory, such that turnaround time for results is often not fast enough to aid in real-time patient management.

In the United States, there are currently marketed DOA/Tox screening immunoassays for 18 targets (i.e., single drugs or drug classes) including: amphetamines, barbiturates, benzodiazepines, cocaine metabolite/benzoylecgonine, buprenorphine, cannabinoids, heroin metabolite/6-acetylmorphine (6-AM), lysergic acid diethylamide (LSD), MDMA/Ecstasy (3,4-methylenedioxymethamphetamine), methadone, methadone metabolite/EDDP (2-ethylidine-1,5-dimethyl-3,3-diphenylpyrrolidine), methaqualone, nicotine metabolite/cotinine, opiates, oxycodone, phencyclidine (PCP), propoxyphene, and TCAs. For some drugs or metabolites (e.g., buprenorphine, heroin metabolite/6-AM), there may be only one or two manufacturers marketing an assay whereas for more common tests (e.g., amphetamines, benzodiazepines, opiates), there are many different marketed assays. Different assays for the same analyte may vary in terms of analytical sensitivity and specificity, leading to potential difficulties in clinical interpretation. DOA/Tox screening test is most often performed on urine but, in some cases, serum/plasma or saliva may be used [5,7,10].

DOA/Tox screening immunoassays may be designed by raising antibodies against a single drug or drug metabolite ('target compound'). Alternatively, multiple target compounds may be used to achieve broader detection of a class of drugs. There is a general trend towards use of monoclonal antibodies in marketed assays, but assays using polyclonal antibodies are still used widely in some cases [6,7]. Theoretically, use of monoclonal antibodies provides more consistent performance over polyclonal antibodies. DOA/Tox screening assays may be directed at classes of drugs such as amphetamines, barbiturates, benzodiazepines, cannabinoids, and opiates [7,10]. In these 'broad specificity' DOA/Tox assays, ideally the specificity of the assay is broad enough to detect a range of 'within-class' compounds but not too non-specific to cross-react with 'out-of-class' compounds that may have similar chemical structures. Other DOA/Tox screening assays are directed towards detection of a single target compound (drug or drug metabolite) without cross-reactivity with other similar structures. Examples of 'single target' DOA/Tox assays include those for buprenorphine, methadone, and propoxyphene.

DOA/Tox screening immunoassays have two main limitations. First, false positives may occur when an 'out-of-class' compound with structural similarity to the target compound(s) causes a positive screening result [3,5,6,10]. Such cross-reactive molecules can be structurally related drugs, drug metabolites, or endogenous compounds [7,11]. Manufacturers of DOA/Tox screening immunoassays typically test commonly used drugs for cross-reactivity including over-the-counter and prescription medications likely to be taken concomitantly with the target drug, as well as various other compounds [12]. Information on assay sensitivity and cross-reactivity is normally reported in the package insert of the assay or the website of the manufacturer. In other cases, cross-reacting compounds for DOA/Tox screening assays are not reported by the assay manufacturer in the package insert but instead are first described in the medical literature. Examples of such published reports of DOA/Tox assay cross-reactivity include fluoroquinolone antibiotic cross-reactivity with opiate assays [13], venlafaxine cross-reactivity with PCP immunoassays [14-16], and quetiapine cross-reactivity with TCA assays [17-19]. The second main limitation of DOA/Tox screening immunaossays is failure to detect some drugs within a class, resulting in false negatives [3,5,6,10]. Examples of false negatives would be inability to detect clonazepam in a benzodiazepines assay or oxycodone in an opiates assay. Some examples of drugs that can cause false negatives and false positives in DOA/Tox immunoassays are listed in Tables 1 and 2.

**Table 1:** Drugs or drug metabolites that can produce false negatives on DOA/Tox screening immunoassays

			**Cross-Reactivity to Marketed Immunoassays**					
**Drug**	**Assay**^1^	**Similarity to assay target compound**^2^	**Abbott**^3^	**Beckman**^3^	**Biosite Triage**^3^	**Microgenics**^3^	**Roche**^3^	**Siemens**^3^

MDMA	AMPH	0.361	1,300	2,500	2,000	1,300	697,000	34,300

Alprazolam	BENZO	0.610	113	300	450	25	219	65

Clonazepam	BENZO	0.656	214	300	350	3,000	307	260

Clonazepam metabolite (7-amino)	BENZO	0.755	2,334	800	7,500	1,000	288	5,700

Clobazam	BENZO	0.796	218	500	700	250	237	260

Buprenorphine	OPIA	0.783			No effect			No effect

Oxycodone	OPIA	0.800	No effect	17,000	20,000	16,000	> 75,000	2,550

Oxymorphone	OPIA	0.847	No effect	No effect	40,000	40,000		> 20,000

Amoxapine	TCA	0.508	No effect		No effect	No effect		No effect

^1 ^Assay abbreviations: AMPH, amphetamines; BENZO, benzodiazepines; OPIA, opiates; TCA, tricyclic antidepressants.

^2 ^Target compounds: AMPH, *d*-amphetamine; BENZO, diazepam; OPIA, morphine; TCA, desipramine. Similarity calculated using MDL public keys with Tanimoto coefficient.

^3 ^Concentration of compound in ng/mL that produces cross-reactivity equal to 1,000 *d*-amphetamine (AMPH assay), 200 ng/mL diazepam (BENZO assay), 300 ng/mL morphine (OPIA assay), or 1,000 ng/mL desipramine (TCA assay). Blank cells indicate that no cross-reactivity data is reported. For all assays except TCA, marketed assays are for Abbott Architect, Beckman, Biosite Triage, Microgenics DRI, Roche cobas c, and Siemens Syva EMIT systems. For TCA, marketed assays are Abbott AxSYM, Biosite Triage, Microgenics DRI serum, and Siemens Syva EMIT systems. See Additional file 1 (tab T) for more details.

**Table 2:** Drugs that can produce false positives on broad specificity DOA/Tox screening immunoassays

			**Cross-Reactivity to Marketed Immunoassays**					
**Drug**	**Assay**^1^	**Similarity to assay target compound**^2^	**Abbott**^3^	**Beckman**^3^	**Biosite Triage**^3^	**Microgenics**^3^	**Roche**^3^	**Siemens**^3^

Phentermine	AMPH	0.778	No effect	No effect	750,000	No effect		25,000

Levofloxacin	OPIA	0.560	1,700,000	No effect	125,000	60,000	200,000	

Dextromethorphan	PCP	0.565	12,900	No effect	500,000	No effect	No effect	12,000

Meperidine	PCP	0.538	34,650	No effect	No effect	No effect	No effect	25,000

Carbamazepine	TCA	0.460	29,972		No effect	No effect		No effect

Cyclobenzaprine	TCA	0.565			2,000	450		

Prochlorperazine	TCA	0.630	999	100,000				

Quetiapine	TCA	0.485	2,484		No effect	No effect		100,000

^1 ^Assay abbreviations: AMPH, amphetamines; OPIA, opiates; PCP, phencyclidine; TCA, tricyclic antidepressants.

^2 ^Target compounds: AMPH, *d*-amphetamine; OPIA, morphine; PCP, phencyclidine; TCA, desipramine. Similarity calculated using MDL public keys with Tanimoto coefficient.

^3 ^Concentration of compound in ng/mL that produces cross-reactivity equal to 1,000 *d*-amphetamine (AMPH assay), 300 ng/mL morphine (OPIA assay), 25 ng/mL phencyclidine (PCP assay), or 1,000 ng/mL desipramine (TCA assay). Blank cells indicate that no cross-reactivity data is reported. For all assays except TCA, marketed assays are Abbott Architect, Beckman, Biosite Triage, Microgenics DRI, Roche cobas c, Siemens Syva EMIT. For TCA, marketed assays are Abbott AxSYM, Biosite Triage, Microgenics DRI serum, and Siemen Syva EMIT. See Additional file 1 (tab T) for more detail.

In clinical practice, drugs are commonly classified by their therapeutic class, but this does not explicitly define how similar drugs may be to one another in terms of chemical structure and their potential for cross-reactivity in DOA/Tox screening immunoassays. Therefore, we have utilized a computational method known as similarity analysis between molecules [20,21]. Variables that can be included in similarity calculations are extensive and include those related to molecular structure, electrostatic potential, shape, and electron density. Similarity analysis has been used widely in the pharmaceutical industry as a 'virtual' screen for identifying drug-like molecules and predicting drug toxicity, and can be valuable in narrowing the number of compounds subjected to *in vitro*, animal, or human testing [20,22,23]. In our analysis, we have used two-dimensional (2D) similarity with the Tanimoto coefficient, which compares two compounds and generates a similarity measure that ranges from 0 to 1, with 0 being maximally dissimilar and 1 being maximally similar [21,24]. We have found that this similarity measure correlates well with cross-reactivity of immunoassays used clinically for DOA/Tox screening and therapeutic drug monitoring [25,26].

In this study, we applied similarity analysis as a quantitative tool to rationalize false positives and false negatives of DOA/Tox screening assays. We have also compiled historical data on prescription drug usage in the United States to demonstrate how changing patterns of drug use may influence clinical utility of DOA/Tox screening assays. Lastly, we present the results of our own investigation into the causes for positive screening results for PCP and TCA screening assays in our medical center, which has adult and pediatric EDs that serve as a regional toxicology referral center.

## Methods

### Similarity Calculations

Similarity searching uses the 'find similar molecules by fingerprints' protocol in the library analysis module of Discovery Studio 2.0 (Accelrys, San Diego, CA). The MDL public keys are a fingerprint which uses a pre-defined set of definitions related to structural features [27]. A fingerprint is created based on pattern matching of the structure to this set of 166 keys. These MDL keys are used separately with the Tanimoto similarity coefficient and an input query molecule [21] and will be referred to as 'Tanimoto similarity'. It should be noted that this type of similarity algorithm does not recognize differences between stereoisomers (e.g., *d*- and *l*-amphetamine or their racemic mixture; citalopram and escitalopram). Sdf files of the structures of the database compounds are available on request from the authors.

### Cross-Reactivity Testing and Confirmatory Testing

Quetiapine fumarate was obtained from Sequoia Research Products (Pangbourne, United Kingdom). Quetiapine *S*-oxide, 7-hydroxyquetiapine, and 11-piperazin-1yl-dibenzo [b, f] [1, 4] thiazepine dihydrochloride (DBTP) were purchased from Molcan (Toronto, Ontario, Canada). These three quetiapine metabolites were tested for cross-reactivity with two different TCA screening immunoassays: (1) Emit^® ^tox™ serum (tricyclic antidepressants) run on Siemens (Dade-Behring) Viva-E analyzers and (2) Biosite Triage^® ^Tox screen. Both assays were performed following manufacturers' instructions on analyzers used for clinical testing. Urine samples were analyzed by GC/MS to identify a wide range of clinically important drugs and drug metabolites by methods previously described [28]. Patient samples from five University of Pittsburgh Medical Center hospitals (Children's, Montefiore, Presbyterian, Shadyside, and Western Psychiatric) showing positive immunoassay screens for PCP or TCAs were followed by GC/MS testing. The studies involving human samples in this report qualified as exempt, and the need for informed written consent was waived, as determined by the University of Pittsburgh Medical Center Institutional Review Board.

### Cross-reactivity data

Cross-reactivity data for DOA/Tox immunoassays were retrieved and compiled from package inserts or the assay manufacturers' websites. Data for levofloxacin in Table 2 was obtained from a published article [13]. Complete data for each assay, with compounds sorted and color-coded by classification, is in Additional file 1 (tabs A-R, T). The most prescribed medications in the United States in 2007 [29] is also included in Additional file 1 (tab S). Information on the most prescribed medications in the United States from 1970–2006 is provided in Additional file 2.

## Results

### Illustration of Molecular Similarity

**Figure 1:**
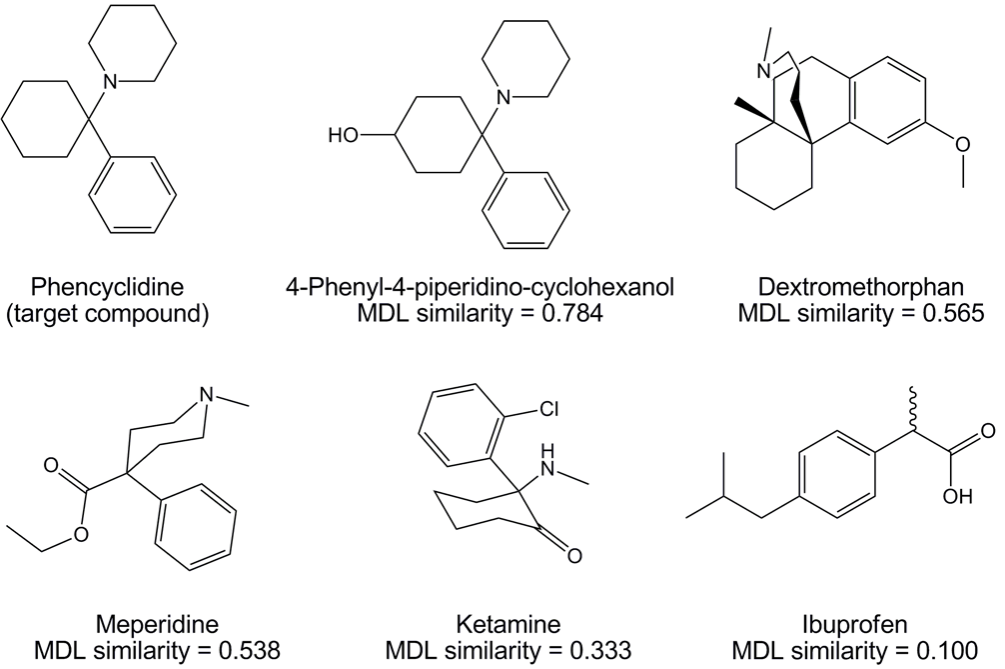
**Illustration of structural similarity**. Using phencyclidine (PCP) as the target compound, 2D similarity was calculated using MDL public keys and the Tanimoto coefficient to five different compounds, three of which (dextromethorphan, meperidine, and the phencyclidine metabolite 4-phenyl-4-piperidino-cyclohexanol) have been reported to cross-react with at least some marketed PCP immunoassays, and two of which (ketamine and ibuprofen) have not been reported to cross-react with PCP screening assays. PCP has the highest similarity (in descending order) to 4-phenyl-4-piperidino-cyclohexanol, dextromethorphan, and meperidine. PCP has low structural similarity to ketamine (despite having similar pharmacological properties to PCP) and essentially no structural similarity to ibuprofen.

We have used the MDL public keys, and a molecular similarity measure (Tanimoto coefficient), to compute the structural similarity between drugs and drug metabolites to the target compounds used in commercially marketed DOA/Tox immunoassay screens. We have illustrated this using PCP as an example. Figure 1 shows the similarity of PCP to 4-phenyl-4-piperidino-cyclohexanol (a PCP metabolite), dextromethorphan (cause of false positives on some PCP screening assays), meperidine (another potential cause of false positives), ketamine, and ibuprofen. PCP has the highest Tanimoto similarity (in descending order) to 4-phenyl-4-piperidino-cyclohexanol (0.784), dextromethorphan (0.565), and meperidine (0.538). All three of these compounds are known to cross-react with some marketed PCP assays, but with varying sensitivities as reported in the package inserts (Additional file 1, tab P).

For example, only 30 ng/mL of 4-phenyl-4-piperidino-cyclohexanol produces cross-reactivity equal to 25 ng/mL PCP in the Abbott Architect PCP assay. Dextromethorphan is reported to cross-react with 4 of 8 commonly marketed PCP assays, with 12,000 ng/mL providing cross-reactivity equal to 25 ng/mL PCP in the Syva EMIT assay but with 500,000 ng/mL needed to do the same for the Biosite Triage assay (Additional file 1, tab P). Meperidine only cross-reacts with 2 of 8 marketed assays (Abbott Architect and Syva EMIT). As shown in Figure 1, ketamine, despite similarity to PCP in terms of mechanism of action and clinical effects [30], is not that closely related to PCP structurally as measured by 2D similarity (0.333) and is also not known to cross-react with marketed PCP assays (Additional file 1, tab P). PCP has essentially no 2D similarity to ibuprofen (0.100), a widely used drug that does not cross-react at all with PCP assays (Figure 1).

We will now apply similarity calculation to our analysis and discussion of individual DOA/Tox screening assays.

### Amphetamine Assays

**Figure 2:**
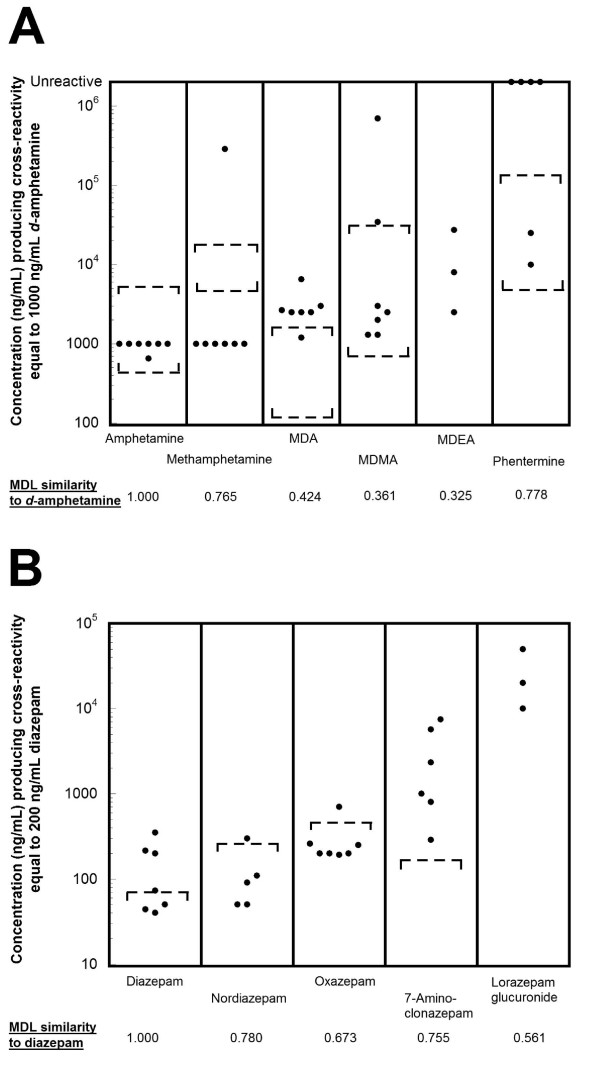
**Variability in sensitivity of marketed amphetamine and benzodiazepine screening immunoassays**. The plotted circles indicate the concentration of compound that produces an equivalent reaction to 1000 ng/mL *d*-amphetamine (amphetamine assays) or 200 ng/mL diazepam (benzodiazepine assays). The dashed lines bracket clinically or toxicologically relevant concentrations from studies in the published literature (see text of Results for detailed description). A) Amphetamine assays. With one exception (Roche cobas c assay), marketed amphetamine screening immunoassays detect amphetamine and methamphetamine well but have variable and often low cross-reactivity with MDA, MDMA, MDEA, and phentermine. B) Benzodiazepine assays. Marketed benzodiazepine screening immunoassays generally have higher sensitivity to diazepam, oxazepam, and nordiazepam than to 7-aminoclonazepam (main clonazepam urinary metabolite) or lorazepam glucuronide (main lorazepam urinary metabolite).

Currently marketed amphetamine screening immunoassays in the United States use *d*-amphetamine, *d*-methamphetamine, or both drugs as the antigenic targets (Additional file 1, tab T). *d*-Amphetamine and *d*-methamphetamine have high similarity to one another (Tanimoto similarity = 0.765) and only 1 currently marketed amphetamine screening assay (Roche cobas c) has markedly different sensitivities for these two amphetamines (Figure 2A; Additional file 1, tab A). There is much more variability in detection by these assays for amphetamine derivatives such as MDMA/Ecstasy (Tanimoto similarity to amphetamine = 0.361) and 3,4-methylenedioxyamphetamine (MDA; Tanimoto similarity to amphetamine = 0.424). The low levels of 2D structural similarity of MDA and MDMA to amphetamine (or methamphetamine) are comparable or lower than those between amphetamine and bupropion (Tanimoto similarity = 0.321), ephedrine (Tanimoto similarity = 0.391), labetalol (Tanimoto similarity = 0.298), mexiletine (Tanimoto similarity = 0.500), phentermine (Tanimoto similarity = 0.778), and pseudoephedrine (Tanimoto similarity = 0.391).

This presents a difficult challenge in developing antibodies broad enough to detect a range of amphetamine derivatives but avoiding widely used drugs with potential for cross-reactivity such as bupropion, labetalol, or pseudoephedrine. Figure 2A shows the cross-reactivities of six marketed amphetamine assays for *d*-amphetamine, *d*-methamphetamine, MDA, MDMA, 3,4-methylenedioxyethylamphetamine (MDEA), and phentermine. As can be seen, there is wide variability in the ability of these assays to detect MDA, MDMA, and MDEA (note the ordinate in Figure 2A is on a logarithmic scale). One clinical consequence of this may be that a patient abusing MDMA can have opposing test results if evaluated by two different assay systems (e.g., because of transfer from one hospital to another). More recently, specific MDMA immunoassays that have good cross-reactivity with MDA and MDEA but essentially no cross-reactivity with *d*-amphetamine or *d*-methamphetamine have been developed and marketed (Additional file 1, tab T).

An additional challenge in interpreting amphetamine screening assay results is that prescriptions for amphetamine mixed salts (e.g., Adderall^®^) are now common, ranking #66 in total volume of prescriptions in the United States in 2007 (Additional file 1, tab S; Table 3). A pharmacokinetic study of individuals taking Adderall^® ^for at least 5 consecutive days showed peak urine concentrations (5,739 to 19,172 ng/mL) that greatly exceed the 1,000 ng/mL cutoff often used in screening immunoassays, and in general urine amphetamine concentrations that were mostly above 1,000 ng/mL [31].

**Table 3:** Frequency of prescriptions of target compounds and structurally related drugs for DOA/Tox immunoassays

**Immunoassay**	**Target compound(s) of marketed assays**	**Highest rank of most prescribed in the United States during 1970s and 1980s**^1^	**Rank as most prescribed in United States in 2007**^2^	**Structurally related top prescribed drugs in 2007 and their rank as most prescribed in United States**^2^
Amphetamines	*d*-Amphetamine *d*-Methamphetamine^3^	Top 100 (1970–1971) Top 200 (1970)	66 Unranked	Bupropion (44)

Barbiturates	Secobarbital	Top 50 (1970–1971)	Unranked	Butalbital (163) Phenobarbital (204)

Benzo-diazepines	Diazepam Nordiazepam^3^ Oxazepam^3^	1 (1972–1979) Not applicable 93 (1976)	71 Not applicable Unranked	Alprazolam (16) Lorazepam (40) Clonazepam (45) Temazepam (111)

Opiates	Morphine	Top 200 (1970)	230	Hydrocodone (2) Oxycodone (17) Codeine (37) Buprenorphine (248) Hydromorphone (270)

Tricyclic antidepressants	Desipramine Imipramine	Unranked 82 (1972)	Unranked 268	Cyclobenzaprine (47) Amitriptyline (70) Quetiapine (92) Nortriptyline (194) Doxepin (236) Carbamazepine (237) Prochlorperazine (240)

^1 ^Top prescribed medications in the United States compiled from multiple sources (see Additional file 2).

^2 ^See Additional file 1, tab T.

^3 ^*d*-Methamphetamine is not prescribed but is widely abused. Nordiazepam is not a prescribed medication but is a metabolite of several benzodiazepines (chlordiazepoxide, clorazepate, diazepam, and prazepam). Oxazepam is both a parent drug and potential metabolite of multiple benzodiazepines (chlordiazepoxide, clorazepate, diazepam, prazepam, and temazepam). Imipramine is metabolized to desipramine.

The plots in Figure 2A include dashed brackets to indicate clinically or toxicologically relevant urine concentrations from published studies. For amphetamine and methamphetamine, the urinary concentrations indicated by dashed brackets in Figure 2A are the ranges found in a pharmacokinetic study involving four consecutive daily doses of methamphetamine [32]. In this study, the urine concentrations achieved generally exceeded the 1,000 ng/mL positive cutoff for both amphetamine and methamphetamine. For MDMA and MDA, the range of urine concentrations indicated by dashed brackets in Figure 2A are from 25 antemortem urine concentrations in fatal cases associated with MDMA overdose [33]. Note that even these very high MDMA and MDA urine concentrations do not exceed the threshold for positivity on some amphetamine screening immunoassays. The dashed brackets in the phentermine plot in Figure 2A are the range of urine concentrations reported in forensic studies of phentermine overdose [34]. These very high phentermine urine concentrations would exceed the positive cutoff for only two marketed amphetamine screening immunoassays. Consequently, currently marketed amphetamine screening immunoassays generally do not cross-react with phentermine or do so only when this drug is taken in extreme overdose.

### Barbiturate Assays

All currently marketed barbiturate immunoassays use secobarbital as a target compound, with some containing antibodies raised only against secobarbital, while others use antibodies raised against multiple barbiturates (Additional file 1, tab T). The choice of secobarbital as the antigenic target in first-generation barbiturate immunoassays followed from this intermediate-acting barbiturate being one of the most heavily prescribed and abused barbiturates of the 1960s and 1970s [35]. Based on similarity calculations, clinically used barbiturates do not possess as much 'within-class' structural variability as the amphetamines discussed above. Clinically important barbiturates have MDL similarities of 0.7 or greater to one another and low structural similarity to other classes of drugs, probably explaining why barbiturate assays have very few documented out-of-class cross-reactive compounds (Additional file 1, tab B). One known cross-reactive drug, aminoglutethimide (Tanimoto similarity = 0.567 relative to secobarbital), is not widely used in the United States and would be an uncommon cause of a barbiturate screening assay false positive. Prescriptions and abuse of barbiturates have been declining steadily in the United States for the past three decades [3]. For example, in the 1970s, six barbiturates were among the most highly prescribed medications in the United States (Additional file 2, figure S2-A). However, other medications such as benzodiazepines, eszopiclone, and zolpidem have steadily replaced barbiturates as safer hypnotics, anxiolytics, and sedatives (Additional file 2-figures S2-B,C). Currently, only two barbiturates (butalbital and phenobarbital) rank in the top most prescribed medications in the United States (Additional file 1, tab S and Additional file 2, figure S2-C) [29].

### Benzodiazepine Assays

First-generation benzodiazepine screening assays of the 1970s used one of three antigenic targets [36,37] – diazepam, nordiazepam, or oxazepam – with a recent shift towards using multiple benzodiazepines as antigenic targets (Additional file 1, tab T). The original choice of diazepam, nordiazepam, or oxazepam also followed from historical trends in usage of benzodiazepines. Diazepam was the most prescribed medication overall in the United States for over a decade (Additional file 2). Other commonly prescribed benzodiazepines of the 1970s, including chlordiazepoxide and clorazepate, are metabolized to nordiazepam and oxazepam (Additional file 2, figure S2-D). Using diazepam, nordiazepam, or oxazepam as target compounds thus fit the prescribing patterns of the 1970s well, either by targeting the most commonly prescribed benzodiazepine of that time (diazepam) or targeting metabolites common to multiple benzodiazepines.

However, three benzodiazepines (alprazolam, clonazepam, and lorazepam) are currently more commonly prescribed in the United States than diazepam (Additional file 2, figure S2-C; Table 3) [29]. None of these three 'newer' benzodiazepines are metabolized to nordiazepam or oxazepam; in addition, each has lower similarity to diazepam than does nordiazepam (Tanimoto similarities to diazepam: nordiazepam, 0.780; lorazepam, 0.673; clonazepam, 0.656; alprazolam, 0.610). The marketed benzodiazepine screening immunoassays therefore have difficulty in detection of clonazepam and lorazepam usage, as compared to the use of diazepam or other early generation benzodiazepines.

Figure 2B plots the cross-reactivities of marketed benzodiazepine assays towards diazepam, nordiazepam, oxazepam, 7-aminoclonazepam, and lorazepam glucuronide (note that cross-reactivity is not reported for all of these compounds for some of the assays). The upper brackets for diazepam, nordiazepam, and oxazepam in Figure 2B indicate the maximum urine concentrations detected in individual consuming a single diazepam dose of 10 mg or less [38]. As can be seen, even a single diazepam dose can result in urine concentration of diazepam and multiple metabolites that exceed the positive cutoff for benzodiazepine screening immunoassays (Figure 2B). Detection would be predicted to be even easier in patients on chronic therapy, where steady-state urine concentrations of diazepam and multiple metabolites would accumulate to even higher concentrations.

Currently marketed benzodiazepine screening assays have limited sensitivity to detecting use of clonazepam. Although marketed benzodiazepine assays have reasonably good sensitivity to clonazepam (parent drug), sensitivity is much lower to the major urinary metabolite 7-aminoclonazepam (Additional file 1, tab C). Following oral administration, the majority of clonazepam appears in the urine as the metabolite 7-aminoclonazepam [39,40]. In a study of clonazepam pharmacokinetics following a single 3 mg dose [40], the highest peak urine concentration of 7-aminoclonazepam (~ 183 ng/mL) recorded in all study participants would still be below the reported concentrations of 7-aminoclonazepam necessary to produce a positive screening result in all currently marketed benzodiazepine screening immunoassays (dashed bracket in Figure 2B; Additional file 1, tab C). Even with chronic administration of clonazepam, urine concentrations of 7-aminoclonazepam may still be below the positive cutoff for most benzodiazepine screening immunoassays in clinical situations.

Currently marketed benzodiazepine screening assays also have difficulty in detecting the use of lorazepam. Studies of lorazepam pharmacokinetics following oral or parenteral administration show that very little unchanged drug is excreted in the urine, with the majority appearing as the glucuronide metabolite [41,42]. Lorazepam glucuronide has low structural similarity to diazepam (Tanimoto similarity = 0.561) and is detected much more poorly by the marketed assays than unconjugated lorazepam (Additional file 1, tab C). Some marketed benzodiazepine immunoassays can include a separate step to cleave the glucuronide bonds (e.g., by enzymatic or chemical reaction), resulting in unconjugated drugs. For a drug such as lorazepam, where the glucuronide metabolite is the predominant form in the urine, cleaving the glucuronide bonds would be predicted to enhance the detection rate. Some marketed assays (e.g., Syva EMIT-H^® ^and Roche Online KIMS^®^) incorporate a glucuronide cleavage step in the reaction, while still maintaining rapid analysis times [43,44].

### Cocaine assays

**Figure 3:**
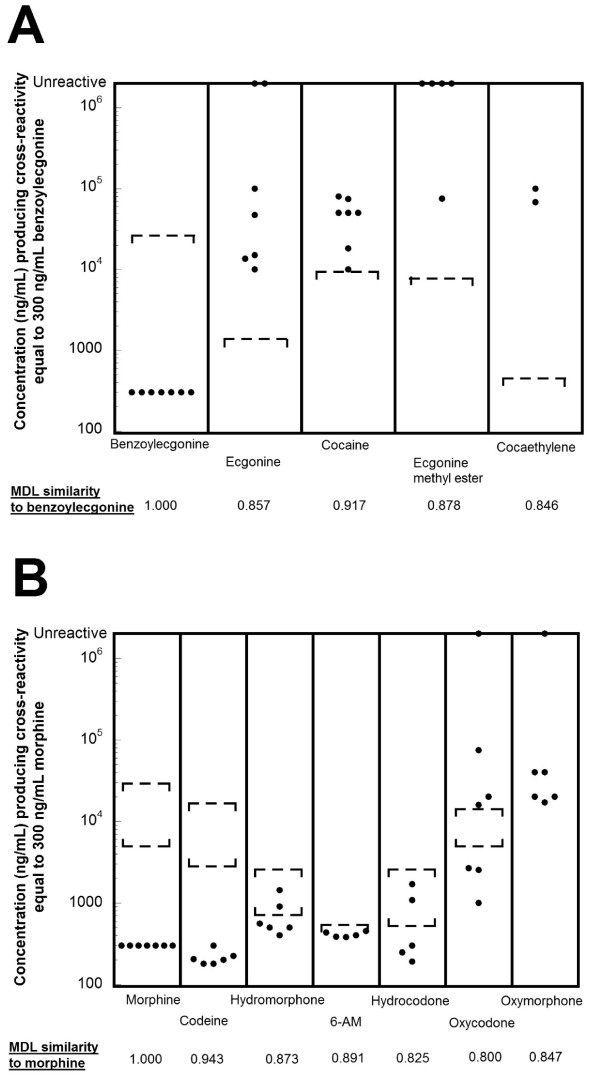
**Variability in sensitivity of marketed cocaine metabolite and opiate screening immunoassays**. The plotted circles indicate the concentration of compound that produces equivalent reaction to 300 ng/mL benzoylecgonine (cocaine metabolite assays) or 300 ng/mL morphine (opiate assays). The dashed lines bracket clinically or toxicologically relevant concentrations from studies in the published literature (see text of Results for detailed description). A) Cocaine metabolite assays. Marketed cocaine metabolite detect benzoylecgonine with high sensitivity but generally have low sensitivity for detection of cocaine (parent drug) and metabolites other than benzoylecgonine. B) Opiate assays. Marketed opiate assays detect morphine, codeine, and hydrocodone well but have variability and often poor sensitivity to oxycodone and oxymorphone.

All cocaine screening immunoassays currently marketed in the United States use antibodies raised against benzoylecognine, one of the two major cocaine metabolites in humans [7], as the antigenic target (Additional file 1, tab T). Thus, the marketed assays can be termed more precisely 'cocaine metabolite screening assays' or 'benzoylecgonine screening assays'. Currently these marketed assays detect cocaine (parent drug) weakly, with cross-reactivities equal to 300 ng/mL benzoylecgonine only occurring at cocaine concentrations ranging from 10,000 ng/mL (Abbott AxSYM) to 80,000 ng/mL (Syva EMIT) (Additional file 1, tab F; Figure 3A). In clinical practice, this means that very recent use of cocaine, even in large amounts, may fail to trigger a positive screen if too little time has elapsed for the metabolism of the parent drug to benzoylecgonine to occur. The marketed assays also vary in detection of other cocaine metabolites such as ecgonine, ecgonine methyl ester (the second major cocaine metabolite in most individuals) [7], and benzylnorecgonine (Additional file 1, tab F; Figure 3A), potentially leading to different results if a patient sample is tested on more than one immunoassay system. The upper brackets for all compounds in Figure 3A except cocaethylene indicate peak urine concentrations of cocaine and metabolites following controlled administration of 40 mg cocaine by the inhalation route [45]. For cocaethylene (an adduct product of cocaine and ethanol), the upper bracket indicates the peak urine concentration in a controlled study of simultaneous cocaine and ethanol administration [46]. Figure 3A illustrates the low cross-reactivity of marketed cocaine metabolite immunoassays to all compounds except benzoylecgonine.

Molecular similarity also explains the generally low false positive rates for cocaine metabolite assays. In terms of similarity, benzoylecgonine has low structural similarity (Tanimoto similarity of 0.615 or lower) to common medications or illicit drugs. Despite being a local anesthetic (in addition to its other effects), cocaine has low structural similarity to other clinically important local anesthetics (e.g., bupivacaine, lidocaine, and procaine), which have Tanimoto similarities to benzoylecgonine of 0.377 or lower, effectively explaining why such compounds or their metabolites do not cause false positives on the currently available cocaine metabolite screening immunoassays (Additional file 1, tab F).

### Opiate assays

Unlike barbiturate and benzodiazepine screening immunoassays, where some manufacturers have used multiple drugs or drug metabolites as antigenic targets, all currently marketed opiate immunoassays use antibodies raised solely against morphine (Additional file 1, tab T). Based on our similarity calculations, this strategy would be predicted to be effective for the sensitive detection of opiates and metabolites that are structurally very close to morphine, including codeine (Tanimoto similarity to morphine = 0.943), heroin (Tanimoto similarity = 0.857), 6-AM (main metabolite of heroin; Tanimoto similarity = 0.891), and hydromorphone (Tanimoto similarity = 0.873). In fact, the package insert data reveals this generally to be true with few exceptions (e.g., a few assays are less sensitive to hydromorphone than to codeine; Additional file 1, tab N; Figure 3B). Currently marketed opiate immunoassays perform less well in their detection of oxycodone (Tanimoto similarity to morphine = 0.800) with 2 of 8 marketed assays being essentially insensitive to oxycodone and 3 additional assays only producing cross-reactivity equal to 300 ng/mL morphine at oxycodone concentrations of 16,000 ng/mL or greater (Additional file 1, tab N; Figure 3B; Table 1). These assays also respond weakly to oxymorphone (Figure 3B), itself a clinically used drug and also a main metabolite of oxycodone [47]. The lack of sensitivity of marketed opiate assays to oxycodone is a serious limitation as this drug is now prescribed more often in the United States than codeine, morphine, and propoxyphene (Additional file 2, figure S2-E; Table 3) and is also a frequently abused prescription medication [48].

The upper and lower brackets for morphine, codeine, hydromorphone, hydrocodone, and oxycodone in Figure 3B represent estimated steady-state urine concentrations in a 70 kg individual during chronic administration of daily 60 mg oral dose of codeine, 10 mg intramuscular dose of morphine, 5 mg oral dose of hydromorphone, 10 mg oral dose of hydrocodone, or 20 mg oral dose of oxycodone, respectively [49]. As can be seen in Figure 3B, the steady-state urine concentrations for all drugs except oxycodone will generally exceed the cutoff equivalent to 300 ng/mL morphine. For oxycodone, only 3 of 7 marketed assays have sensitivities to oxycodone sufficient to readily detect daily use of 20 mg oral oxycodone. Exact urine concentrations of oxymorphone following either oxymorphone or oxycodone administration have not been reported in the literature but are likely to be well below the assay sensitivities due to the extensive metabolism of oxymorphone prior to renal excretion [50,51]. The upper bracket for 6-AM is the highest peak 6-AM urine concentration observed in a study of controlled heroin administration [52].

Marketed opiate assays do not cross-react with the mixed opiate agonist-antagonist buprenorphine (Tanimoto similarity to morphine = 0.783) (Additional file 1, tab N). Commonly used non-opiate opioid drugs (e.g., fentanyl, meperidine, methadone, propoxyphene) generally have low structural similarity to morphine (Tanimoto similarity range = 0.407 – 0.522) and either do not cross-react, or do so only at extremely high concentrations, with opiate screening immunoassays (Additional file 1, tab N).

### Phencyclidine assays

**Figure 4:**
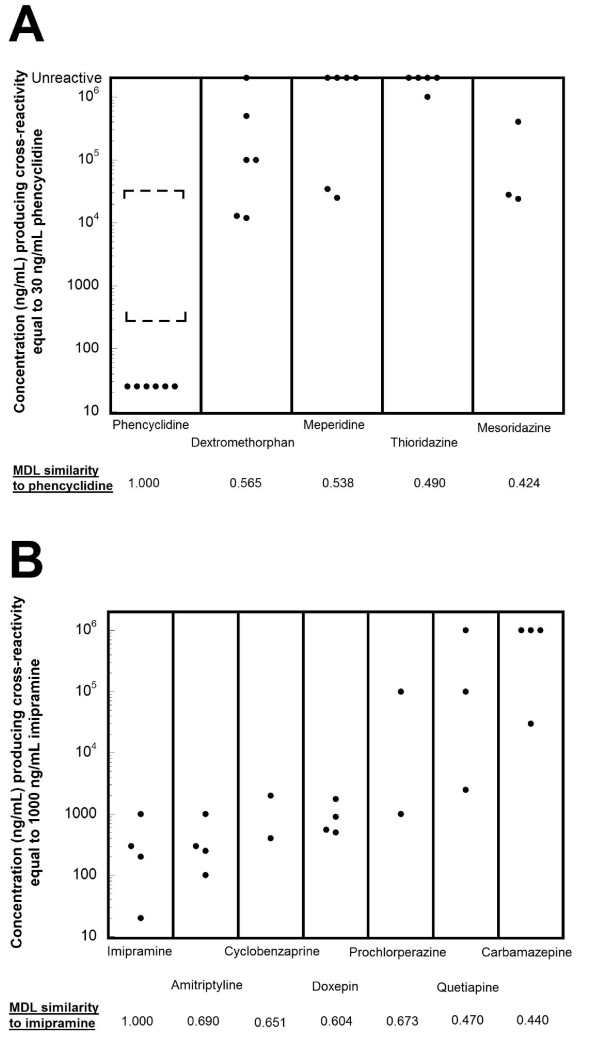
**Variability in sensitivity of marketed PCP and tricyclic antidepressant screening immunoassays**. The plotted circles indicate the concentration of compound that produces equivalent reaction to 25 ng/mL PCP or 1000 ng/mL desipramine (tricyclic antidepressant assays). The dashed lines bracket clinically or toxicologically relevant concentrations from studies in the published literature (see text of Results for detailed description). A) PCP assays. Marketed PCP assays have varying degrees of cross-reactivity with dextromethorphan, meperidine, thioridazine, and mesoridazine. The brackets for PCP correspond to urine concentrations observed in patients abusing PCP[54] B) TCA assays. Marketed TCA screening immunoassays have similar cross-reactivities to TCAs but variable cross-reactivity to carbamazepine, phenothiazines (such as prochlorperazine), and quetiapine. The marketed TCA assays include those approved for serum/plasma or urine samples.

As a drug of abuse in the United States, PCP has waxed and waned in popularity over time, with substantial regional differences in usage of this drug [53]. There are five well-documented cross-reactive compounds with PCP immunoassays: dextromethorphan, venlafaxine [14-16], meperidine, thioridazine, and mesoridazine (Additional file 1, tab P), although high urine concentrations of these drugs are generally required to elicit a positive PCP screening result (Figure 4A; brackets indicate urine concentrations of PCP in patients abusing PCP [54]). While prescriptions for the latter three drugs have declined in the United States over the last decade, venlafaxine is widely prescribed in the United States (55^th ^most prescribed drug in 2008) [29], and dextromethorphan continues to be widely used as both a prescription and over-the-counter medication in anti-tussive remedies (Table 3) [29]. Supra-therapeutic doses of dextromethorphan-containing medications are sometimes abused for psychoactive effects, most frequently by adolescents and young adults [55].

In our own medical system, we had anecdotally observed that PCP abuse by patients presenting to our ED was rare, whereas intentional dextromethorphan and meperidine usage was more common, suggesting that there may be many false positive PCP screening tests. We therefore examined the causes of PCP positive screens in our medical system (Additional file 1, tab V). Over the course of 24 months (January 2007 through January 2009), we had 10 patient samples with positive PCP screens, nine on the Syva EMIT assay and only one on the Biosite Triage assay system (Additional file 1, tab V). Urine samples from these 10 patients were also analyzed by GC/MS, using a protocol to definitively identify a wide range of clinically important legal and illicit drugs [28]. Of these, only one patient showed the definitive presence of PCP by GC/MS (30 year old female); this positive screen occurred on the Syva EMIT system. The one positive PCP screen on the Biosite Triage assay in our sample occurred in a 48 year old female, with GC/MS analysis showing apparently very high urine concentrations of diphenhydramine, a medication reported to cause positives on the Biosite Triage PCP assay at high urine concentrations (Additional file 1, tab P). The eight patient samples (average age 23.6 years, range 2–44 years old; 5 males, 3 females) that had a positive PCP screen on the Syva EMIT assay not accounted for by the presence of PCP all showed apparently high urine concentrations of dextromethorphan by GC/MS that would be consistent with intentional or inadvertent overdose of dextromethorphan. One of these samples also showed the presence of apparently high urine concentrations of meperidine by GC/MS. Relative to PCP, dextromethorphan (Tanimoto similarity = 0.565) and meperidine (Tanimoto similarity = 0.538) have similarity coefficients higher than some of the PCP metabolites compared to their parent drug (Additional file 1, tab P).

The common misuse of dextromethorphan suggests that, in medical settings where PCP use is uncommon, false positives on some marketed PCP assays (e.g., Syva EMIT) due to dextromethorphan can occur more frequently than true positives. In these cases, PCP screening assays may become more effective as 'dextromethorphan overdose screens' than as PCP screens. However, it should be pointed out that at least five of the currently marketed PCP immunoassays are reported to be insensitive to dextromethorphan (Additional file 1, tab P; Figure 4A). The Biosite Triage system used in some hospitals in our medical system is an example of this. This may explain why all but one of the PCP screen positives we documented occurred on the Syva EMIT system, for which only 12,000 ng/mL of dextromethorphan, a concentration easily obtainable in patients taking dextromethorphan-containing medications in overdose [55,56] will trigger a screen positive equal to 25 ng/mL PCP (Additional file 1, tab P). It should be noted that our laboratory has, over the last two years, analyzed many urine specimens where dextromethorphan is detectable by GC/MS but the PCP screen is negative, supporting that only high urine concentrations of dextromethorphan can cause a false positive PCP screen.

### Tricyclic Antidepressant Assays

Currently marketed TCA screening immunoassays use either desipramine or imipramine, or multiple TCAs, as target compounds (Additional file 1, tab T). Our similarity calculations indicate that screening for TCAs is a difficult challenge for an immunoassay. In particular, several phenothiazines and other non-TCA drugs have a relatively high structural similarity to desipramine (or other TCAs), which may explain why some non-TCA compounds cross-react well with TCA screening assays (Additional file 1, tab R; Figure 4B) [57-61]. Examples of the Tanimoto similarities of TCAs and other tricyclic compounds relative to desipramine are: amitriptyline (0.600), carbamazepine (0.460), chlorpromazine (0.630), cyclobenzaprine (0.565), doxepin (0.529), nortriptyline (0.628), prochlorperazine (0.630), and quetiapine (0.485) (Table 3).

**Figure 5:**
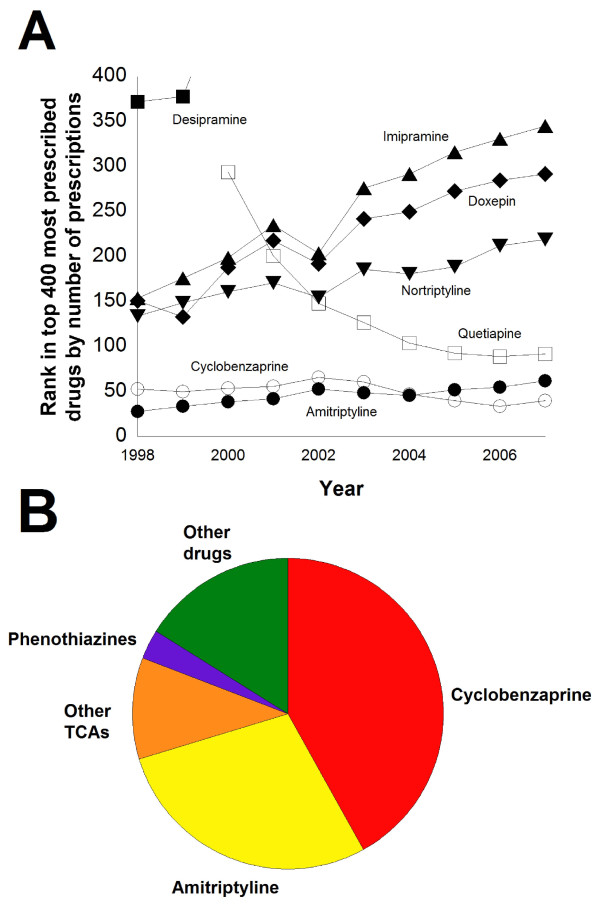
**Tricyclic antidepressant assays**. A) Rank of tricyclic antidepressants, cyclobenzaprine, and quetiapine by total number of prescriptions in the United States in the time period from 1998–2007. TCAs are indicated by closed symbols, while the non-TCAs (cyclobenzaprine and quetiapine) are designated by open circles and squares, respectively. Whereas prescriptions for amitriptyline have remained relatively constant in the last decade, prescriptions for other TCAs are steadily declining, with desipramine no longer ranking in the top 400 most prescribed drugs. Cyclobenzaprine is now prescribed more frequently than amitriptyline in the United States. B) Drugs most likely accounting for positive TCAs immunoassay screens in our medical center sample. Of 124 positive TCA screens (see Additional file 1, tab U for details), the most likely causes were sorted into five categories: cyclobenzaprine, amitripytline +/- nortriptyline, other TCAs (e.g. doxepine, imipramine, and their metabolites), phenothiazines, and other drugs (e.g., carbamazepine and quetiapine).

An additional challenge for TCA screening assays is that prescriptions for TCAs have declined markedly in the United States in the last fifteen years as other medications such as the selective serotonin reuptake inhibitors (SSRIs) have assumed steadily increasing shares of the market for treatment of depression, obsessive-compulsive disorder, and other psychiatric conditions (Additional file 2, figures S2-G,H) [62]. This is illustrated in Figure 5A which shows the rank of TCAs, cyclobenzaprine, and quetiapine among the top prescribed medications in the United States in the time period from 1998 to 2007. In 2007 [29], only amitriptyline (#70) ranked in the top 100 most prescribed medications, likely due in part to the extensive use of amitriptyline for treating chronic pain [63], whereas nine non-TCA antidepressants rank in the top 100 most prescribed medications (sertraline, #23; escitalopram, #26; fluoxetine, #36; bupropion, #44; paroxetine, #49; venlafaxine, #55; citalopram, #56; trazodone, #59, and duloxetine, #79) (Additional file 1, tab S; Additional file 2, figures S2-G,H). As shown in Figure 5A, cyclobenzaprine was prescribed more often than amitriptyline in 2007, and quetiapine has also been approaching amitriptyline in total number of prescriptions. Meanwhile, prescriptions for the TCAs desipramine, doxepin, imipramine, and nortriptyline have steadily declined in the last decade with desipramine no longer ranking in the top most prescribed medications (Figure 5A, Additional file 1, tab S). Prescriptions for phenothiazines have also declined dramatically over the last decade in the United States, with 'atypical' antipsychotics increasing significantly in clinical use (Additional file 2, figures S2-H,I). This decline in use of phenothiazines means they will likely account for increasingly fewer false positives on TCA screening assays.

In our own medical system, we use two different assay methods for TCA screening (Biosite Triage^® ^and Syva Emit^® ^serum tox™). Over the course of 24 months (January 2007 through January 2009), we performed GC/MS analysis for the broad detection of drugs and drug metabolites on all samples that returned a positive screening result for TCA assays (109 on Triage and 15 on Syva) to determine the most likely cause of the positive result (Additional file 1, tab U). As shown in Figure 5B, 70% of the positive results were accounted for by the presence of amitriptyline (28.2%) or cyclobenzaprine (41.9%), a centrally acting muscle relaxant that differs from amitriptyline by the presence of one double bond [64]. Other TCAs accounted for 10.5% (1 imipramine and 10 doxepin cases) of the positive screens while two phenothiazines (chlorpromazine and prochlorperazine) accounted for only 3.2% of the positive screens. Thus, TCAs only accounted for 38.7% of the most likely causes for the positive screening results in our study. The remaining positive screens (16.0% of the total) included patients whose urine showed high concentrations of venlafaxine (n = 2), carbamazepine (n = 2), topiramate (n = 1), or quetiapine (n = 1). The frequency of drugs most likely causing positive TCA screens in our sample fits well with the overall prescription trends in the United States (Figure 5A; Additional file 1, tab S) and the known cross-reactivities of the Biosite Triage and Syva EMIT assay systems. For example, the package insert for the Triage assay states that a cyclobenzaprine urine concentration of only 2000 ng/mL will produce cross-reactivity equal to 1000 ng/mL TCAs (Additional file 1, tab R).

Given the current widespread use of cyclobenzaprine in the United States (Figure 5A) [29,64], it is not surprising to encounter false positive TCA screening assay results due to the presence of this drug. The increasing use of cyclobenzaprine and quetiapine will likely result in more and more TCA positive screens resulting from these compounds [17-19,59,65], especially combined with increasing reports of intentional misuse and overdoses with quetiapine [66-68].

Previous studies have shown cross-reactivity of quetiapine (parent drug) with marketed TCA assays but did not look at quetiapine metabolites [17-19,59,65]. We therefore tested the Syva EMIT and Biosite Triage TCA screening assays with drug-free serum (Syva) or urine (Biosite) spiked with pure reference standards of each of three quetiapine metabolites (DBTP, quetiapine *S*-oxide, 7-hydroxyquetiapine) or quetiapine itself. None of these four compounds, even at very high concentrations, cross-reacted with the Biosite assay. We did find, however, that cross-reactivity equal to 1000 ng/mL desipramine in the Syva assay was produced by 100,000 ng/mL quetiapine, 50,000 ng/mL DBTP, or 200,000 ng/mL quetiapine *S*-oxide. Although to our knowledge there is no published data on serum or urine concentrations of quetapine metabolites following quetiapine overdose, our data suggest that quetiapine metabolites may contribute to cross-reactivity with some TCA screening immunoassays.

### DOA/Tox Screening Assays to Address Limitations of Standard Assays

As we have seen, several broad-specificity DOA/Tox immunoassays may fail to detect all clinically important members of a class of drugs (Table 1). To address this issue, manufacturers have developed and marketed assays for buprenorphine, heroin metabolite/6-AM, MDMA, and oxycodone (Additional file 1, tabs D, H, J, and O). The currently marketed assays for buprenorphine and oxycodone are reported to be highly specific for only these drugs and their main metabolites (i.e., buprenophine glucuronide and oxymorphone, respectively) [69]. One possible limitation of the oxycodone assay is that it will not distinguish between the use of either oxycodone and oxymorphone. The only currently marketed heroin metabolite immunoassay cross-reacts well with heroin and weakly with structurally related opiates (hydromorphine, morphine) (Additional file 1, tab H). The only currently marketed MDMA assay cross-reacts well with a number of designer amphetamines that are related structurally, e.g, MDA (Tanimoto similarity to MDMA = 0.889) and MDEA (0.850) but essentially does not cross-react with the less similar *d*-amphetamine (0.361) or *d*-methamphetamine (0.457) (Additional file 1, tab J).

## Discussion

DOA/Tox screening immunoassays are widely used in emergency medicine [5,7,10]. These assays are also used by substance abuse treatment centers, chronic pain clinics, and psychiatric units, in addition to employee and competitive athlete drug screening programs [5,10]. The multiple uses of DOA/Tox screening tests probably provides substantial inertia to attempts to alter assay design and performance, as changes in assay design and detection cutoffs could have wide-ranging impacts. The most common set of DOA/Tox screening assays (e.g., amphetamines, barbiturates, benzodiazepines, cocaine metabolite, opiates, and PCP), and their antigenic targets, have remained remarkably similar across the last four decades.

As we have shown, false negatives for DOA/Tox screening assays aimed at drug classes can occur with drugs that became widely used in clinical practice in the United States after the 1970s and which have relatively low structural similarity to the classic antigenic targets of their associated immunoassays. For benzodiazepines, this includes alprazolam, clobazam, clonazepam, and lorazepam, while for opiates this includes buprenorphine, oxycodone, and oxymorphone. A few marketed immunoassays (e.g., Biosite Triage) have attempted to broaden specificity by using antibodies raised against multiple antigenic targets. The potential disadvantage of this approach is reduced specificity and increased false positives. Also, any alteration of these immunoassays has implications for workplace and athlete testing, leading to pressure to keep assay performance stable across many years of testing.

Many marketed DOA/Tox screening immunoassays have documented cross-reactive drugs that can produce false positives. In the medical setting, false positives can lead to incorrect diagnoses and treatment. One way to limit false positives is to use higher concentration cutoffs for determining what constitutes a positive screening result, although this has the trade-off of reducing sensitivity. This strategy is common in workplace DOA testing where cutoff concentrations for a variety of DOA screening tests are often higher than cutoffs used in the medical setting, so as to limit false positives that require costly and time-consuming confirmatory testing [7,10]. For example, using higher cutoffs helps reduce the issue of poppy seed ingestion causing a positive opiate screen [70] or passive marijuana inhalation resulting in a tetrahydrocannabinol positive screen [71].

We demonstrated that PCP and TCA screening assays are prone to false positives by common drugs that may be taken in overdose, either in suicide attempts or for psychotropic effects (e.g., dextromethorphan, meperidine). In our own medical center study, there were more false positives than true positives for both PCP and TCA screening assays applied to a clinical sample that included many ED patients. This brings into question the utility of these particular tests in settings where use/abuse of the target drug(s) is uncommon.

One application of our Tanimoto similarity assessment using the MDL keys would be to identify compounds that have a high likelihood of cross-reacting with marketed immunoassays. The 2D similarity method can readily screen very large databases of many thousands of drugs (including herbal products) and their metabolites. Compounds with high Tanimoto similarity to the immunoassay antigenic target(s) can then be prioritized for testing for cross-reactivity. This approach would provide a more systematic approach to cross-reactivity testing and may identify previously unknown clinically important cross-reactive drug or drug metabolites more quickly, leading to an increased recognition of potential cross-reactivity by clinicians.

The steady increase in prescription and over-the-counter medications available clinically presents a difficult challenge for future DOA/Tox immunoassay design. Some newer therapeutic classes of drugs that are often taken in overdose (e.g., atypical antipsychotics, SSRI antidepressants) are actually not that closely related to one another (see Additional file 1, tabs W and X). This is a contrast to older drug classes (e.g., barbiturates, benzodiazepines, and TCAs), where there is significant structural similarity between all drugs within the class. The clinical implication of this is that it would be difficult to design an 'SSRI overdose screen' or 'atypical antipsychotic overdose screen' with standard immunoassay technology. It also suggests that development of DOA/Tox immunoassays has not kept pace with the development of new drugs relevant to the ED community or with changes in patterns of abuse of illicit and prescription drugs.

The analytical methods currently used mainly for DOA/Tox confirmatory testing, such as GC/MS and liquid chromatography/tandem mass spectrometry (LC/MS/MS), can specifically identify (and in some cases quantitate) drugs and their metabolites. This technology, however, is technically demanding, labor-intensive, expensive compared to immunoassays, and usually available only at reference laboratories or in clinical laboratories associated with larger medical centers [7]. A future goal would be to develop and adapt GC/MS, LC/MS/MS, or a novel technology in a manner to be more widely accessible clinically, so as to provide detailed drug exposure data with a rapid turnaround time, allowing ED physicians to make more specific diagnoses and treatment plans. A scientifically similar challenge is in emerging technology to develop portable yet analytically robust sensors for chemical warfare agents or environmental pollutants [72], and there may be opportunities to develop clinical applications using related technology.

An important limitation of the 2D similarity approach used in our study is that this cannot account for the complex three-dimensional (3D) molecular interactions that mediate antibody-antigen binding as occurs in immunoassays. To our knowledge, a 3D structure of an antibody used in a marketed DOA/Tox screening immunoassay bound to its antigenic target has not been reported, although there has been structural determination of several other antibodies being evaluated as novel antidotes to DOA overdose (e.g., PCP [73] and cocaine [74,75]), in which the antibody interacts with all portions of the target molecule. For DOA/Tox screening immunoassays where similar antibody-drug interactions apply, whole molecule similarity measures (as used in our study) seem appropriate for prediction; however, this may not always be the case. A crystal structure of morphine bound to a monoclonal antibody showed that the antibody interacted with the more hydrophobic portion of morphine, while the hydrophilic half was mostly solvent exposed [76]. For target compounds like morphine, similarity searching using substructures may therefore be worth evaluating, although depending on the size of the molecule, complexity, and novelty this may yield many more molecules predicted as positives.

## Conclusion

A combination of computational molecular similarity and historical data analysis of the number of prescriptions for drugs per year highlights some of the challenges in use of routine DOA/Tox screening immunoassays for patient management in emergency medicine. Although several immunoassays do not currently yield high numbers of false positives, others do and these can be simply explained by some degree of structural similarity with the immunoassay antigen. Additional DOA/Tox immunoassays for some therapeutic drug classes (e.g., benzodiazepines, opiates) possess high levels of false negatives resulting from newer drugs which may have comparatively lower structural similarity with the immunoassay antigenic target. ED physicians should therefore be aware of substantial variability in different marketed assays with respect to cross-reactivity of drugs, metabolites, and natural products. There is a current need for improved immunoassays or novel more specific technologies and closer tracking of prescribing trends for drugs likely to cross-react with DOA/Tox immunoassays.

## Competing interests

The authors declare that they have no competing interests.

## Authors' contributions

MDK conceived of the study and structured the data. MDK and SE drafted the manuscript. AFP participated in the planning of the study, interpretation of data, and the historical data analysis. MGS and SG performed and analyzed the laboratory studies involving immunoassays and GC/MS. SE and MI performed the computational analyses. All authors participated in editing and revising the manuscript and approved the final version.

## Pre-publication history

The pre-publication history for this paper can be accessed here:



## Supplementary Material

Additional file 1 **Similarity data and tricylic antidepressant/phencyclidine assay data**. Spreadsheet with multiple tables contains data on similarity analyses, marketed assays, most prescribed medications, and cross-reactivity studies for phencyclidine and tricyclic antidepressant assays. Click here for file

Additional file 2 **Historical trends in prescription drug usage in the United States that can impact drug of abuse testing**. Data on trends in prescription drugs usage is presented by classes of drugs. Click here for file

## References

[B1] Becker ML, Kallewaard M, Caspers PW, Visser LE, Leufkens HG, Stricker BH (2007). Hospitalisations and emergency department visits due to drug-drug interactions: a literature review. Pharmacoepidemiol Drug Saf.

[B2] D'Onofrio G, Becker B, Woolard RH (2006). The impact of alcohol, tobacco, and other drug use and abuse in the emergency department. Emerg Med Clin North Am.

[B3] Wu AHB, McKay C, Broussard LA, Hoffman RS, Kwong TC, Moyer TP, Otten EM, Welch SL, Wax P (2003). National Academy of Clinical Biochemistry laboratory medicine practice guidelines: recommendations for the use of laboratory tests to support poisoned patients who present to the emergency department. Clin Chem.

[B4] Phillips DP, Barker GE, Eguchi MM (2008). A steep increase in the domestic fatal medication errors with use of alcohol and/or street drugs. Arch Intern Med.

[B5] Hammett-Stabler CA, Pesce AJ, Cannon DJ (2002). Urine drug screening in the medical setting. Clin Chim Acta.

[B6] Kricka LJ, Burtis CA, Ashwood ER, Bruns DE (2006). Principles of immunochemical techniques. Tietz textbook of clinical chemistry and molecular diagnostics.

[B7] Porter WH, Burtis CA, Ashwood ER, Bruns DE (2006). Clinical toxicology. Tietz textbook of clinical chemistry and molecular diagnostics.

[B8] Mulé SJ, Bastos ML, Jukofsky D (1974). Evaluation of immunoassay methods for detection, in urine, of drugs subject to abuse. Clin Chem.

[B9] Spector S, Flynn EJ (1971). Barbiturates: radioimmunoassay. Science.

[B10] Moeller KE, Lee KC, Kissack JC (2008). Urine drug screening: practical guide for clinicians. Mayo Clin Proc.

[B11] Kricka LJ (2000). Interferences in immunoassays – still a threat. Clin Chem.

[B12] Powers DM, Boyd JC, Glick MR (1986). Interference testing in clinical chemistry (EP7-A).

[B13] Baden LR, Horowitz G, Jacoby H, Eliopoulos GM (2001). Quinolones and false-positive urine screening for opiates by immunoassay technology. JAMA.

[B14] Bond GR, Steele PE, Uges DR (2003). Massive venlafaxine overdose resulted in a false positive Abbott AxSYM urine immunoassay for phencyclidine. J Toxicol Clin Toxicol.

[B15] Santos PM, López-García PN, J S, Fernández AS, Sádaba B, Vidal JP (2007). False positive phencyclidine results caused by venlafaxine. Am J Psychiatry.

[B16] Sena SF, Kazimi S, Wu AH (2002). False-positive phencyclidine immunoassay results caused by venlafaxine and *O*-desmethylvenlafaxine. Clin Chem.

[B17] Caravati EM, Juenke JM, Crouch BI, Anderson KT (2005). Quetiapine cross-reactivity with plasma tricyclic antidepressant immunoassays. Ann Pharmacother.

[B18] Henrickson RG, Morocco AP (2003). Quetiapine cross-reactivity among three tricyclic antidepressant immunoassays. J Toxicol Clin Toxicol.

[B19] Sloan KL, Haver VM, Saxon AJ (2000). Quetiapine and false-positive urine drug testing for tricyclic antidepressants. Am J Psychiatry.

[B20] Bender A, Glen RC (2004). Molecular similarity: a key technique in molecular informatics. Org Biomol Chem.

[B21] Willett P (2003). Similarity-based approaches to virtual screening. Biochem Soc Trans.

[B22] Ekins S, Mestres J, Testa B (2007). *In silico *pharmacology for drug discovery: applications to targets and beyond. Br J Pharmacol.

[B23] Reddy AS, Pati SP, Kumar PP, Pradeep HN, Sastry GN (2007). Virtual screening in drug discovery – a computational perspective. Curr Protein Pept Sci.

[B24] Hert J, Willett P, Wilton DJ, Acklin P, Azzaoui K, Jacoby E, Schuffenhauer A (2004). Comparison of topological descriptors for similarity-based virtual screening using multiple bioactive reference structures. Org Biomol Chem.

[B25] Krasowski MD, Siam MG, Iyer M, Ekins S (2009). Molecular similarity methods for predicting cross-reactivity With therapeutic drug monitoring immunoassays. Ther Drug Monit.

[B26] Krasowski MD, Siam MG, Iyer M, Pizon AF, Giannoutsos S, Ekins S (2009). Chemoinformatic methods for predicting interference in drug of abuse/toxicology immunoassays. Clin Chem.

[B27] Paolini GV, Shapland RH, van Hoorn WP, Mason JS, Hopkins AL (2006). Global mapping of pharmacological space. Nat Biotechnol.

[B28] Pizon AF, Schwartz AR, Shum LM, Rittenberger JC, Lower DR, Giannoutsos S, Virji MA, Krasowski MD (2009). Toxicology laboratory analysis and human exposure to *p*-chloroaniline. Clin Toxicol (Phila).

[B29] (2008). Red Book.

[B30] Wolff K, Winstock AR (2006). Ketamine: from medicine to misuse. CNS Drugs.

[B31] Cody JT, Valtier S, Nelson SL (2004). Amphetamine excretion profile following multidose adminstration of mixed salt amphetamine preparation. J Anal Toxicol.

[B32] Kim I, Oyler JM, Moolchan ET, Cone EJ, Huestis MA (2004). Urinary pharmacokinetics of methamphetamine and its metabolite, amphetamine following controlled oral administration to humans. Ther Drug Monit.

[B33] Liu RH, Liu H-C, Lin D-L (2006). Distribution of methylenedioxymethamphetamine (MDMA) and methylenedioxyamphetamine (MDA) in postmortem and antemortem specimens. J Anal Toxicol.

[B34] Levine B, Caplan YH, Dixon AM (1984). A fatality involving phentermine. J Forensic Sci.

[B35] Katz RL (1972). Sedatives and tranquilizers. New Engl J Med.

[B36] Bastiani RJ, Phillips RC, Schneider RS, Ullman EF (1973). Homogenous immunochemical drug assays. Am J Med Technol.

[B37] Peskar B, Spector S (1973). Quantitative determination of diazepam in blood by immunoassay. J Pharmacol Exp Ther.

[B38] Kanto J, Sellman R, Haataja M, Hurme P (1978). Plasma and urine concentrations of diazepam and its metabolites in children, adults and in diazepam-intoxicated patients. Int J Clin Pharmacol Biopharm.

[B39] Kaplan SA, Alexander K, Jack ML, Puglisi CV, de Silva JAF, Lee TL, Weinfeld RE (1974). Pharmacokinetic profiles of clonazepam in dog and humans and flunitrazepam in dog. J Pharm Sci.

[B40] Negrusz A, Bowen AM, Moore CM, Dowd SM, Strong MJ, Janicak PG (2003). Elimination of 7-aminoclonazepam in urine after a single dose of clonazepam. Anal Bioanal Chem.

[B41] Greenblatt DJ, Joyce TH, Comer WH, Knowles JA, Shader RI, Kyriakopoulos AA, MacLaughlin DS, Ruelius HW (1977). Clinical pharmacokinetics of lorazepam. II. Intramuscular injection. Clin Pharmacol Ther.

[B42] Greenblatt DJ, Schillings RT, Kyriakopoulos AA, Shader RI, Sisenwine SF, Knowles JA, Ruelius HW (1976). Clinical pharmacokinetics of lorazepam. I. Absorption and disposition of oral ^14^C-lorazepam. Clin Pharmacol Ther.

[B43] Borrey D, Meyer E, Duchateau L, Lambert W, Van Peteghem C, De Leenheer A (2002). Enzymatic hydrolysis improves the sensitivity of Emit screening for urinary benzodiazepines. Clin Chem.

[B44] Klette KL, Wiegand RF, Horn CK, Stout PR, Magluilo J (2005). Urine benzodiazepine screening using Roche Online KIMS immunoassay with β-glucuronidase hydrolysis and confirmation by gas chromatography-mass spectrometry. J Anal Toxicol.

[B45] Huestis MA, Darwin WD, Shimomura E, Lalani SA, Trinidad DV, Jenkins AJ, Cone EJ, Jacobs AJ, Smith ML, Paul BD (2007). Cocaine and metabolites urinary excretion after controlled smoked administration. J Anal Toxicol.

[B46] Harris DS, Everhart ET, Mendelson J, Jones RT (2003). The pharmacology of cocaethylene administration in humans following cocaine and ethanol administration. Drug Alcohol Depend.

[B47] Chamberlin KW, Cottle M, Neville R, Tan J (2007). Oral oxymorphone for pain management. Ann Pharmacother.

[B48] Compton WM, Volkow ND (2006). Major increases in opioid analgesic abuse in the United States: concerns and strategies. Drug Alcohol Depend.

[B49] Mayo Medical Laboratories, Urine Opiates. http://www.mayomedicallaboratories.com/test-catalog/Clinical+and+Interpretive/8473.

[B50] Cone EJ, Darwin WD, Buchwald WF, Gorodetzky CW (1983). Oxymorphone metabolism and urinary excretion in human, rat, guinea pig, rabbit, and dog. Drug Metab Dispos.

[B51] Pöyhiä R, Seppäla T, Olkkola KT, Kalso E (1992). The pharmacokinetics and metabolism of oxycodone after intramuscular and oral administration to healthy subjects. Br J Clin Pharmacol.

[B52] Smith ML, Shimomura ET, Summers J, Paul BD, Jenkins AJ, Darwin WD, Cone EJ (2001). Urinary excretion profiles for total morphine, free morphine, and 6-acetylmorphine following smoked and intravenous heroin. J Anal Toxicol.

[B53] Thombs DL (1989). A review of PCP abuse trends and perceptions. Public Health Rep.

[B54] Cone EJ, Buchwald W, Yousnefnejad D (1981). Simultaneous determination of phencyclidine and monohydroxylated metabolites in urine of man by gas chromatography-mass fragmentography with methane chemical ionization. J Chromatogr.

[B55] Schwartz RH (2005). Adolescent abuse of dextromethorphan. Clin Pediatr (Phila).

[B56] Bryner JK, Wang UK, Hui JW, Bedodo M, MacDougall C, Anderson IB (2006). Dextromethorphan abuse in adolescence: an increasing trend: 1999–2004. Arch Pediatr Adolesc Med.

[B57] Dasgupta A, Wells A, Datta P (2007). False-positive serum tricyclic antidepressant concentrations using fluorescence polarization immunoassay due to the presence of hydroxyzine and cetirizine. Ther Drug Monit.

[B58] Meenan GM, Barlotta S, Lehrer M (1990). Urinary tricyclic antidepressant screening: comparison of results obtained with Abbott FPIA reagents and Syva EIA reagents. J Anal Toxicol.

[B59] Melanson SE, Lewandrowski EL, Griggs DA, Flood JG (2007). Interpreting tricyclic antidepressant measurements in urine in an emergency department setting: comparison of two qualitative point-of-care urine tricyclic antidepressant drug immunoassays with quantitative serum chromatographic analysis. J Anal Toxicol.

[B60] Nebinger P, Koel M (1990). Specificity data of the tricyclic antidepressants assay by fluorescent polarization immunoassay. J Anal Toxicol.

[B61] Schwartz JG, Hurd IL, Carnahan JJ (1994). Determination of tricyclic antidepressants for ED analysis. Am J Emerg Med.

[B62] Belmaker RH, Agam G (2008). Major depressive disorder. New Engl J Med.

[B63] Jann MW, Slade JH (2007). Antidepressant agents for the treatment of chronic pain and depression. Pharmacotherapy.

[B64] Lofland JH, Szarlej D, Buttaro T, Shermock S, Jalalil S (2001). Cyclobenzaprine hydrochloride is a commonly prescribed centrally acting muscle relaxant, which is structurally similar to tricyclic antidepressants (TCAs), and differs from amitriptyline by only one double bond. Clin J Pain.

[B65] Van Hoey NM (2005). Effect of cyclobenzaprine on tricyclic antidepressant assays. Ann Pharmacother.

[B66] Hussain MZ, Waheed W, Hussain S (2005). Intravenous quetiapine abuse. Am J Psychiatry.

[B67] Reeves RR, Brister JC (2007). Additional evidence of the abuse potential of quetiapine. South Med J.

[B68] Waters BM, Joshi KG (2007). Intravenous quetiapine-cocaine use ("Q-ball"). Am J Psychiatry.

[B69] Backer RC, Monforte JR, Poklis A (2005). Evaluation of the DRI^® ^Oxycodone immunoassay for the detection of oxycodone in urine. J Anal Toxicol.

[B70] Fraser AD, Worth D (1999). Experience with a urine opiate screening and confirmation cutoff of 2000 ng/mL. J Anal Toxicol.

[B71] Mørland J, Bugge A, Skuterud B, Steen A, Wethe GH, Kjeldsen T (1985). Cannabinoids in blood and urine after passive inhalation of Cannabis smoke. J Forensic Sci.

[B72] Arduini F, Amine A, Moscone D, Ricci F, Palleschi G (2007). Fast, sensitive and cost-effective detection of nerve agents in the gas phase using a portable instrument and an electrochemical biosensor. Anal Bioanal Chem.

[B73] Lim K, Owens SM, Arnold L, Sacchettini JC, Linthicum DS (1998). Crystal structure of monoclonal 6B5 Fab complexed with phencyclidine. J Biol Chem.

[B74] Larsen NA, Zhou B, Heine A, Wirsching P, Janda KD, Wilson IA (2001). Crystal structure of a cocaine-binding antibody. J Mol Biol.

[B75] Pozharski E, Moulin A, Hewagama A, Shanafelt AB, Petsko GA, Ringe D (2005). Diversity in hapten recognition: structural study of an anti-cocaine M82G2. J Mol Biol.

[B76] Pozharski E, Wilson MA, Hewagama A, Shanafelt AB, Petsko G, Ringe D (2004). Anchoring a cationic ligand: the structure of the Fab fragment of the anti-morphine antibody 9B1 and its complex with morphine. J Mol Biol.

