# Supplementary material for: Using molecular similarity to highlight the challenges of routine immunoassay-based drug of abuse/toxicology screening in emergency medicine
Source: BMC Emerg Med. 2009 Apr 28;9:5. doi: 10.1186/1471-227X-9-5 (PMC2688477; doi:10.1186/1471-227X-9-5)
Supplement: Additional file 2 — Historical trends in prescription drug usage in the United States that can impact drug of abuse testing. Data on trends in prescription drugs usage is presented by classes of drugs. [file 1471-227X-9-5-S2.pdf]

Additional file 2 - Historical trends in prescription drug usage in the United States that can impact drug of abuse testing

Challenges and limitations of routine immunoassay-based drug of abuse screening used in emergency medicine

Matthew D Krasowski, Anthony F Pizon, Mohamed G Siam, Spiros Giannoutsos, Manisha Iyer, Sean Ekins

Background

Information on prescription drug utilization in the United States in the time period from 1970-2007 is scattered in a number of different publications that present and organize the data in various formats [1-38]. We compiled and analyzed this data focusing on amphetamines, antidepressants, antipsychotics, barbiturates, benzodiazepines, other sedative/hypnotic/anxiolytic drugs, and opioids. Until 1997, the prescription drugs were ranked by number of prescriptions, but the actual volumes were not provided. Therefore, for comparisons across the time period from 1970 to 2007, we simply compared whether drugs were in the top 200 most prescribed drugs. From 1998-2007 [28-38], the data for numbers of prescriptions were provided, allowing for more detailed comparisons. To make the data more relevant to our study, the total number of dispensed prescriptions containing an active drug were determined, combining generic and branded formulations

of the same drug. For combination medications (e.g., hydrocodone/acetaminophen), each active product was counted separately as a drug. This analysis provides a measure of total exposure to drugs. The sorted data for 2007 is found in Additional file 1, tab T.

References

1. Anonymous: **Top 200 drugs in 1970**. *Pharm Times* 1971, **37**(4):29-33.
2. Anonymous: **Top 200 drugs in 1971**. *Pharm Times* 1972, **38**(4):31-35.
3. Anonymous: **Top 200 drugs...new generic Rxs continue to rise in 1973, accounting for 10.6% of new prescriptions**. *Pharm Times* 1974, **40**(4):35-41.
4. Anonymous: **Top 200 drugs...1973 vs. 1974: 4.3% decline refills spawns 0.9% dip in overall Rx volume**. *Pharm Times* 1975, **41**(4):39-46.
5. Anonymous: **Top 200 drugs...1974 vs. 1975: generics rise by 3.2% despite 1% dip in total Rx volume**. *Pharm Times* 1976, **42**(4):37-44.
6. Anonymous: **The top 200 drugs**. *Pharm Times* 1977, **43**(4):37-44.
7. Anonymous: **The top 200 drugs**. *Pharm Times* 1979, **45**(4):29-37.
8. Anonymous: **The top 200 prescription drugs of 1981**. *Am Drug* 1982, **185**(2):20,24-25.
9. Anonymous: **The top 200 prescription drugs of 1982**. *Am Drug* 1983, **187**(2):13,16,19.
10. Anonymous: **The top 200 prescription drugs of 1983**. *Am Drug* 1984, **189**(2):42,47-48,50.
11. Anonymous: **1983 National Prescription Audit: top 200 drugs products account for 61.8% of all Rxs**. *Pharm Times* 1984, **50**(4):27-35.
12. Anonymous: **The top 200 prescription drugs of 1984**. *Am Drug* 1985, **191**(2):30,32,36,40.
13. Anonymous: **The top 200 prescription drugs of 1985**. *Am Drug* 1986, **193**(2):18,21,27,30.
14. Anonymous: **The top 200 drugs of 1985**. *Pharm Times* 1986, **52**(4):25-33.
15. Anonymous: **The top 200 Rx drugs of 1986**. *Am Drug* 1987, **195**(2):19,23,26,28,32.
16. Simonsen LL: **Top 200 drugs of 1987**. *Pharm Times* 1988, **54**(4):38-46.
17. Anonymous: **The top 200 Rx drugs of 1988**. *Am Drug* 1989, **199**(2):38,40,45-46,48,51-52.
18. Anonymous: **Top 200 drugs of 1989**. *Am Drug* 1989, **201**(2):26,28,30,32,34,36,38-39.
19. Simonsen LL: **Top 200 drugs of 1988**. *Pharm Times* 1989, **55**(4):40-48.
20. Anonymous: **The top Rx drugs of 1990**. *Am Drug* 1991, **203**(2):56,58,60,62,65-66,68.
21. **Red Book**. Montvale, NJ: Medical Economics Data Production Company; 1994.
22. **Red Book**. Montvale, NJ: Medical Economics Company, Inc.; 1995.
23. Simonsen LL: **Top 200 drugs**. *Pharm Times* 1995, **61**(4):17-20.

24. **Red Book**. Montvale, NJ: Medical Economics Company, Inc.; 1996.
25. Anonymous: **Top 200 drugs**. *Am Drug* 1996, **215**(2):18-20,23-24,26.
26. **Red Book**. Montvale, NJ: Medical Economics Company, Inc.; 1997.
27. Anonymous: **Top 200 drugs 1996**. *Pharm Times* 1997, **62**(4):27,29-30,32-34,36.
28. **Red Book**. Montvale, NJ: Medical Economics Company, Inc.; 1998.
29. **Red Book**. Montvale, NJ: Medical Economics Company, Inc.; 1999.
30. **Red Book**. Montvale, NJ: Medical Economics Company, Inc.; 2000.
31. **Red Book**. Montvale, NJ: Medical Economics Company, Inc.; 2001.
32. **Red Book**. Montvale, NJ: Thompson Medical Economics, Inc.; 2002.
33. **Red Book**. Montvale, NJ: Thompson PDR; 2003.
34. **Red Book**. Montvale, NJ: Thompson PDR; 2004.
35. **Red Book**. Montvale, NJ: Thomson PDR; 2005.
36. **Red Book**. Montvale, NJ: Thompson PDR; 2006.
37. **Red Book**. Montvale, NJ: Thompson PDR; 2007.
38. **Red Book**. Montvale, NJ: Thomson Healthcare; 2008.

A

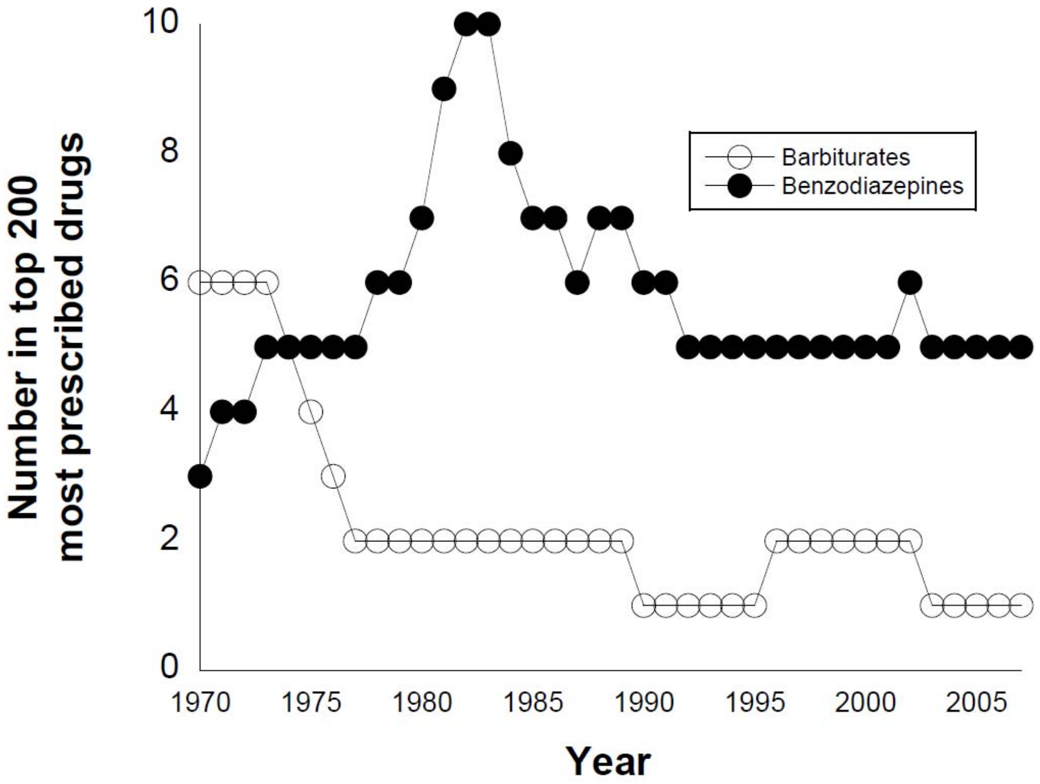

Figure S2-A. Number of barbiturates and benzodiazepines among the top 200 most prescribed medications in the United States in the time period from 1970 to 2007. The barbiturates were the dominant sedative/hypnotics until the mid-1970s but were then displaced by benzodiazepines. Currently, only one barbiturate (butalbital, found in various combination products) is in the top 200 most prescribed medications.

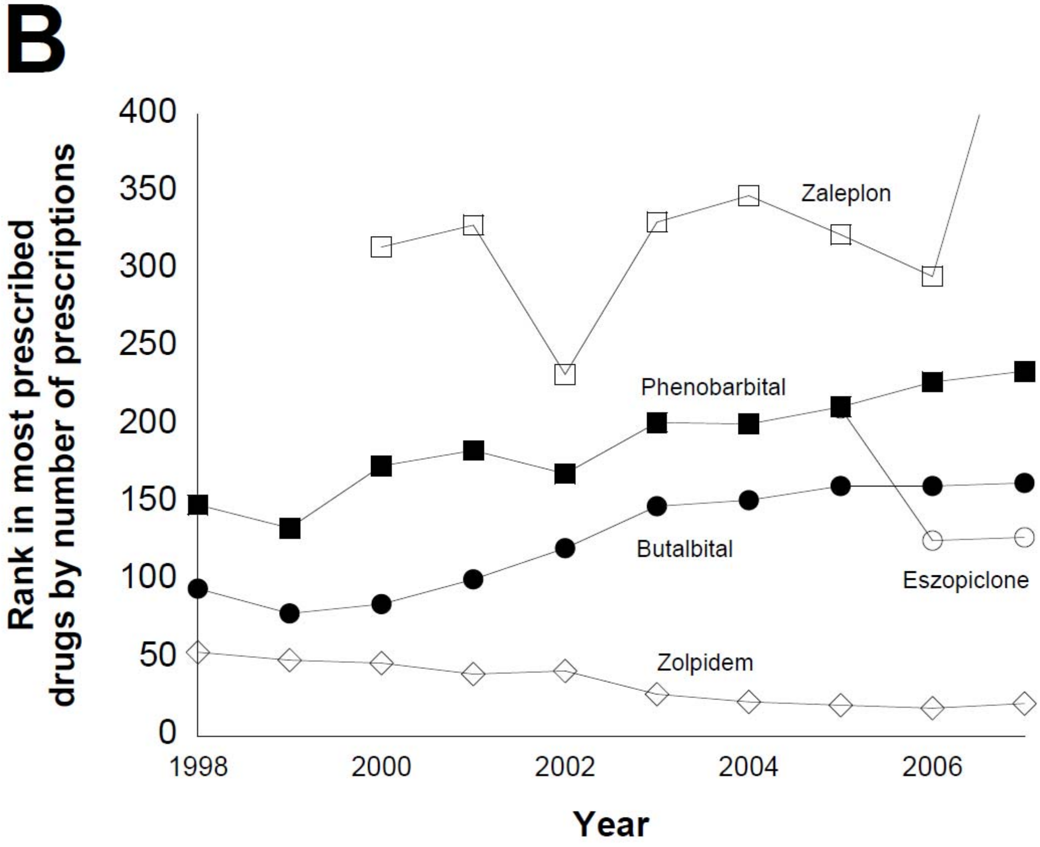

Figure S2-B. Rank of two barbiturate (●, ■) and three non-barbiturate/non-benzodiazepine hypnotic medications (○, ◊, □) by total number of prescriptions in the United States in the time period from 1998-2007. In the last decade, prescriptions for butalbital and phenobarbital have been steadily declining, while prescriptions for eszopiclone and zolpidem (two drugs not detected by any currently marketed DOA/Tox immunoassay screen) are increasing.

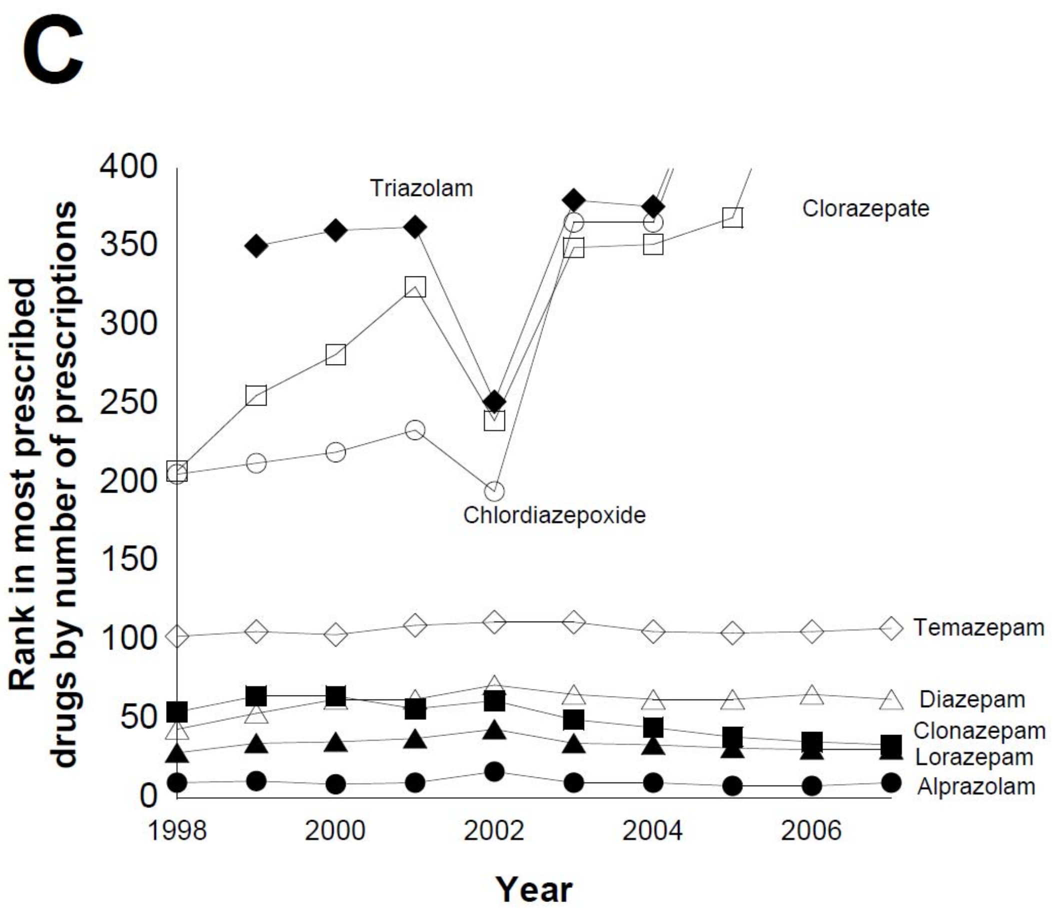

Figure S2-C. Rank of benzodiazepine medications by total number of prescriptions in the United States in the time period from 1998-2007. Prescriptions for chlordiazepoxide, clorazepate, and triazolam have declined to the point that these drugs are no longer ranked in the top 400 most prescribed medications. Prescriptions for five other benzodiazepine medications (alprazolam, clonazepam, diazepam, lorazepam, and temazepam) have been relatively steady over the last decade. 'Classic' benzodiazepines that share metabolites with diazepam (and are generally best detected by currently marketed benzodiazepine screening immunoassays) are indicated by open symbols. All other benzodiazepines are indicated by closed symbols.

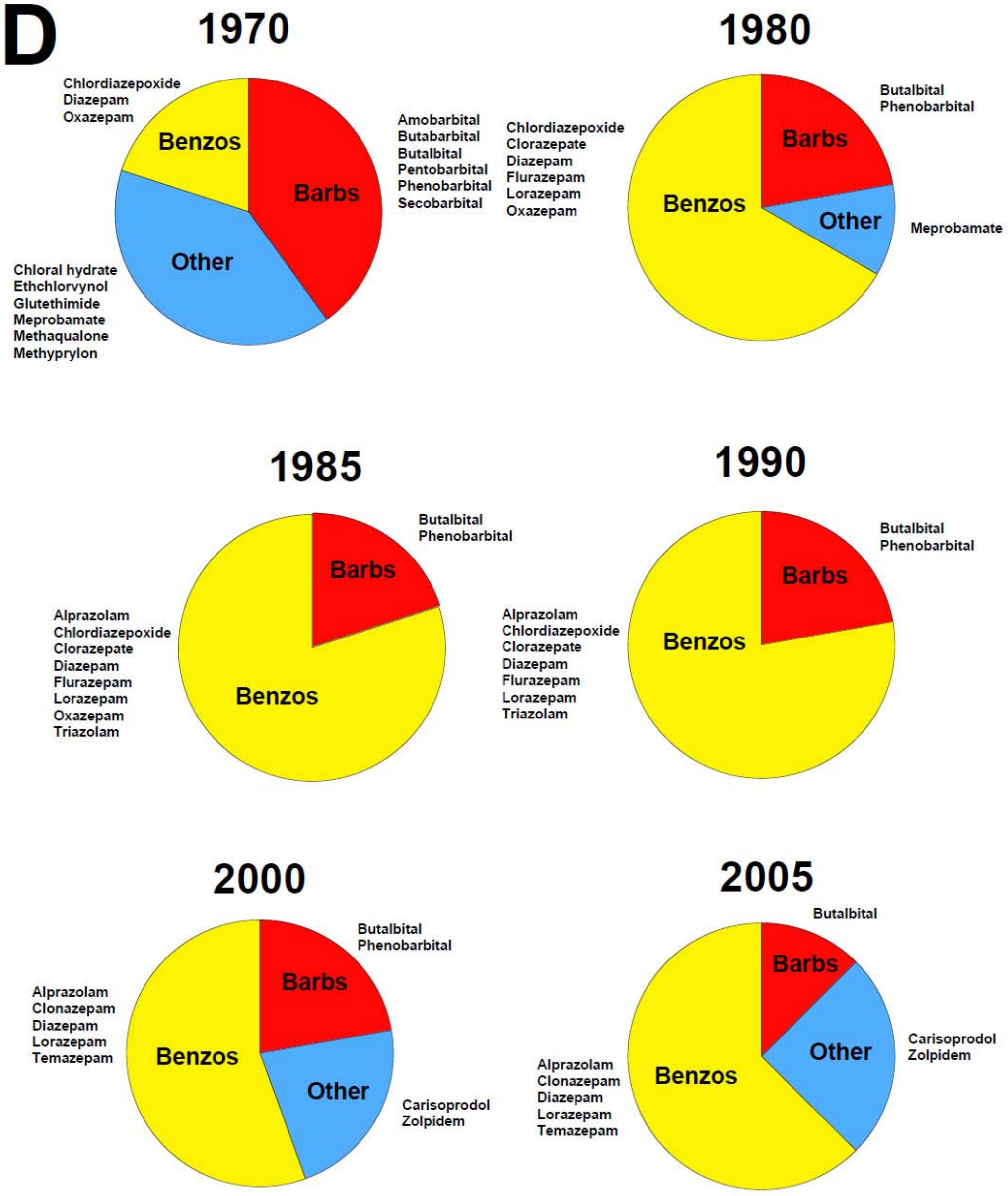

Figure S2-D. Shifts in number of prescriptions for barbiturate, benzodiazepine, and other sedative/hypnotic/anxiolytic medications in the United States in the time period from 1970-2007. The pie charts indicate number of drugs in each of three categories – barbiturates (barbs), benzodiazepines (benzos), and other – that were ranked in the top 200 most prescribed medications in six different years.

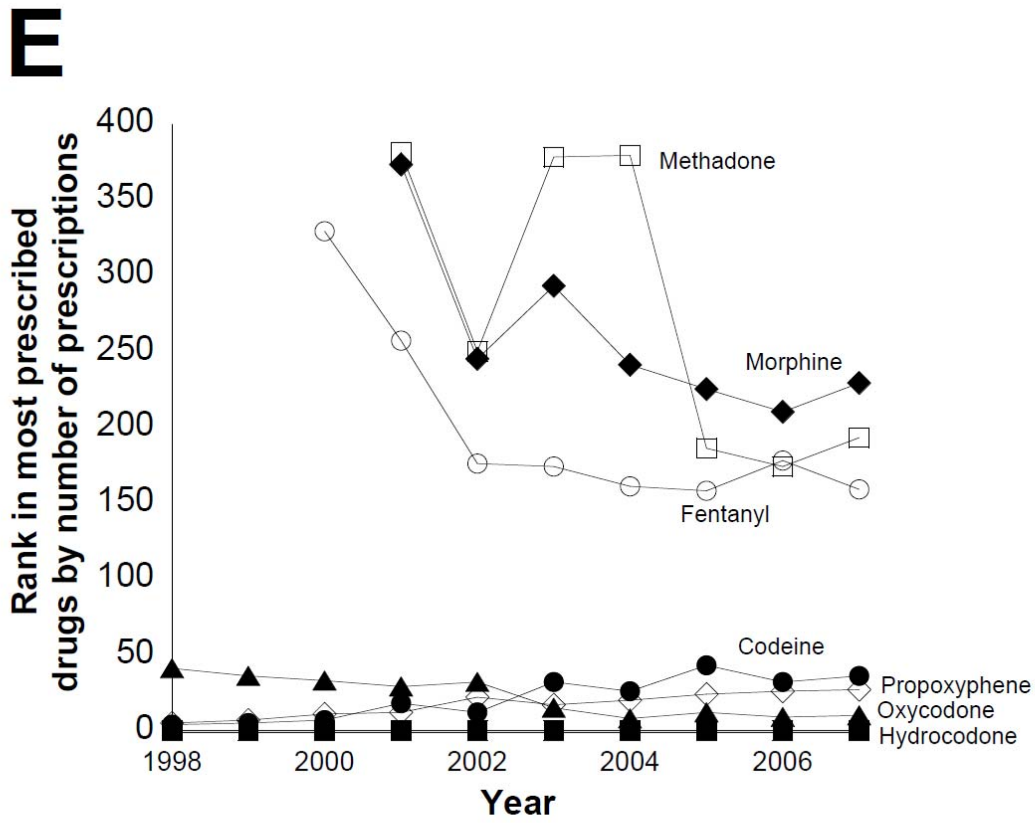

Figure S2-E. Rank of opioid medications by total number of prescriptions in the United States in the time period from 1998-2007. Opiates are indicated by closed symbols. Non-opiate opioids (fentanyl, methadone, and propoxyphene) are indicated by open symbols. Prescriptions for codeine, hydrocodone, and oxycodone have remained in the top 50 most prescribed medications and relatively steady in number over the last decade. Prescriptions for fentanyl, methadone, and morphine have increased in number during the last seven years.

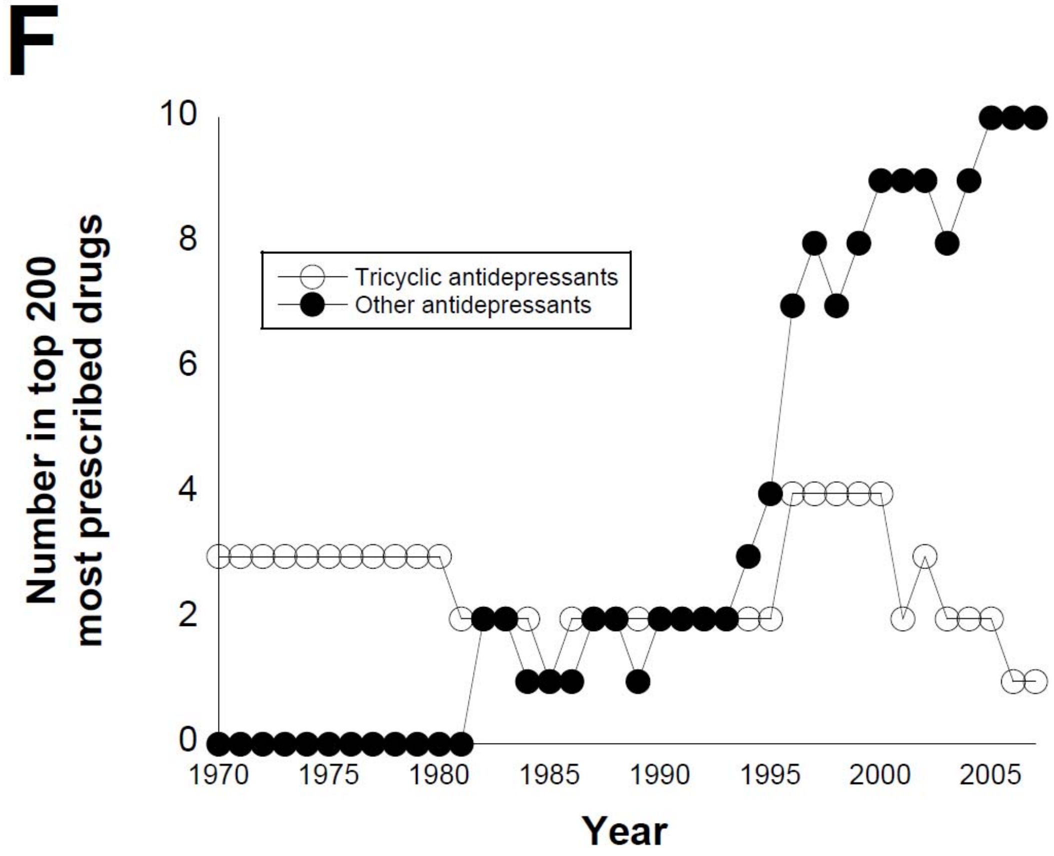

Figure S2-F. Number of antidepressants among the top 200 most prescribed medications in the United States in the time period from 1970-2007. The antidepressants are divided into two broad categories: tricyclic antidepressants (○, includes amitriptyline, desipramine, doxepin, imipramine, and nortriptyline) and other antidepressants (●, includes selective serotonin reuptake inhibitors, serotonin-norepinephrine inhibitors, bupropion, and trazodone). Currently, only one tricyclic antidepressant (amitriptyline) is in the top 200 most prescribed medications in the United States. Cyclobenzaprine, commonly prescribed as a muscle relaxant, was not included as a tricyclic antidepressant for this figure, although this drug is structurally very closely related to amitriptyline and other tricyclic antidepressants.

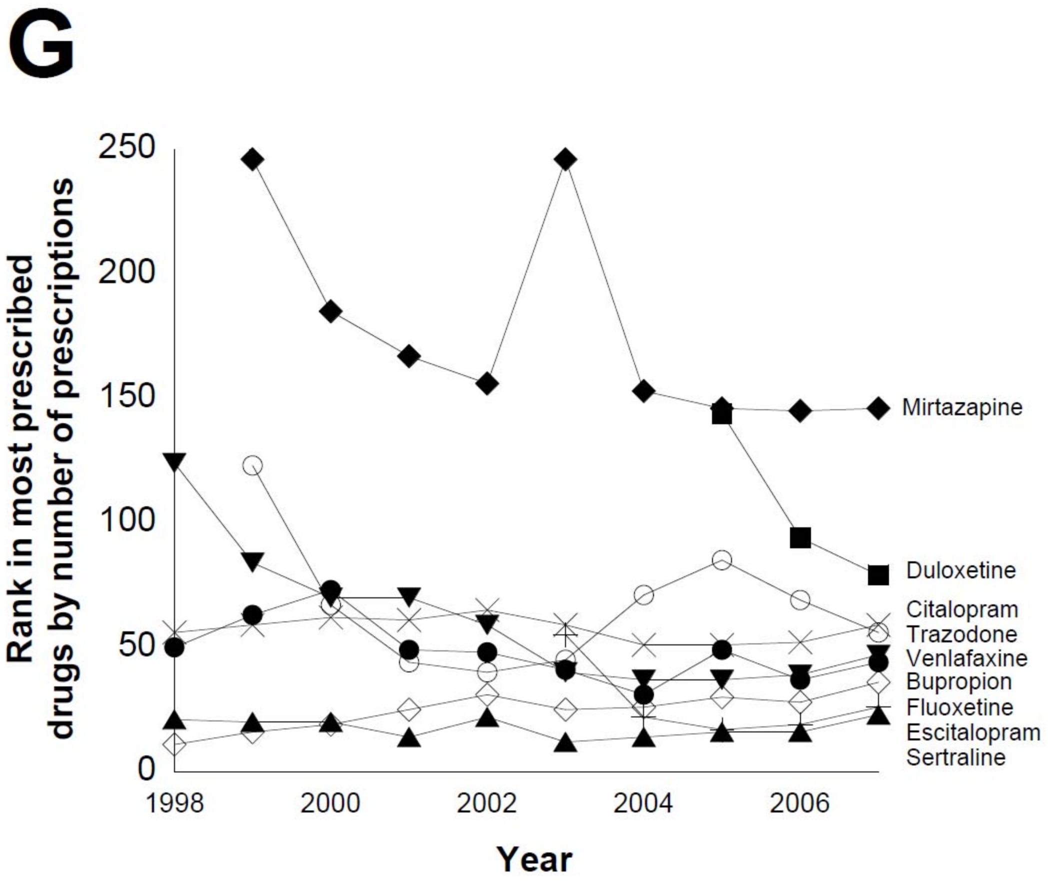

Figure S2-G. Rank of non-tricyclic antidepressant medications by total number of prescriptions in the United States in the time period from 1998-2007. Eight of these antidepressant medications rank in the top 80 most prescribed medications. A similar plot that includes tricyclic antidepressants is found in the main body of the manuscript (Figure 5A).

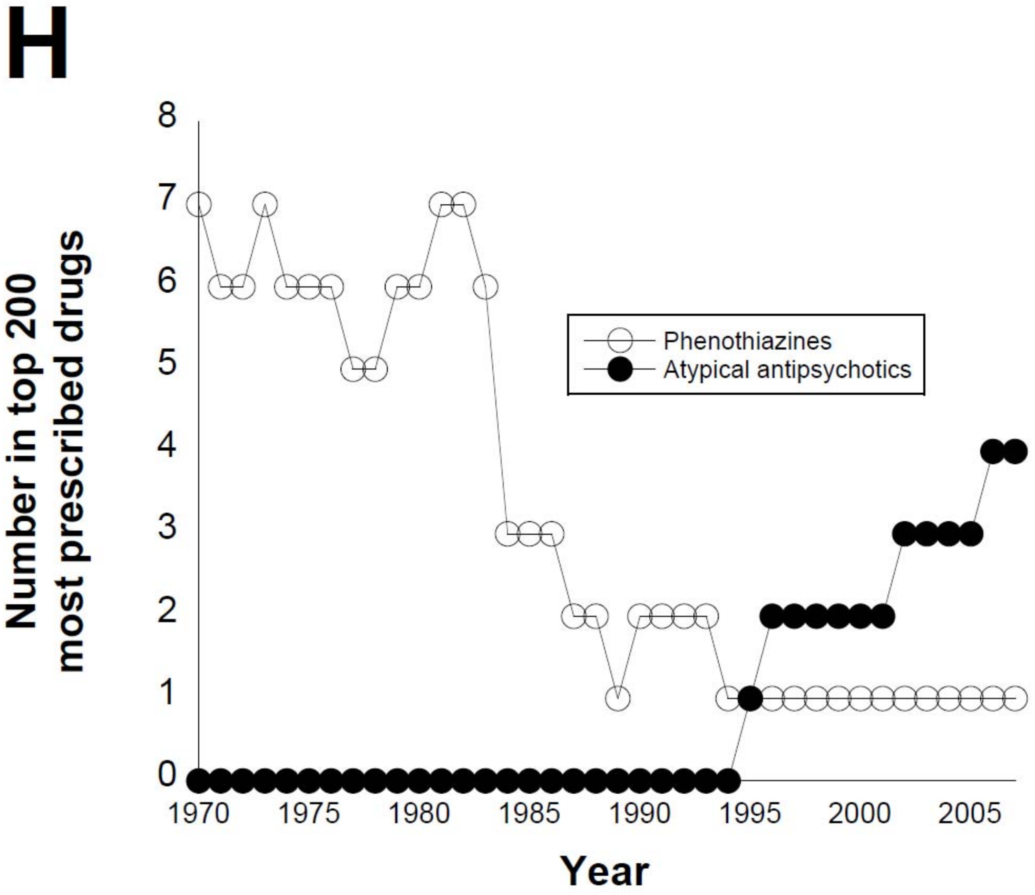

Figure S2-H. Number of antipsychotics among the top 200 most prescribed medications in the United States in the time period from 1970-2007. The antipsychotics are divided into two broad categories: phenothiazines (○, includes a variety of drugs such as chlorpromazine, thioridazine, and trifluoperazine) and atypical antipsychotics (●, includes aripiprazole, clozapine, olanzapine, quetiapine, and risperidone). Note that some phenothiazines, particularly prochlorperazine and promethazine, are likely most widely used for indications other than psychotic illness.

I

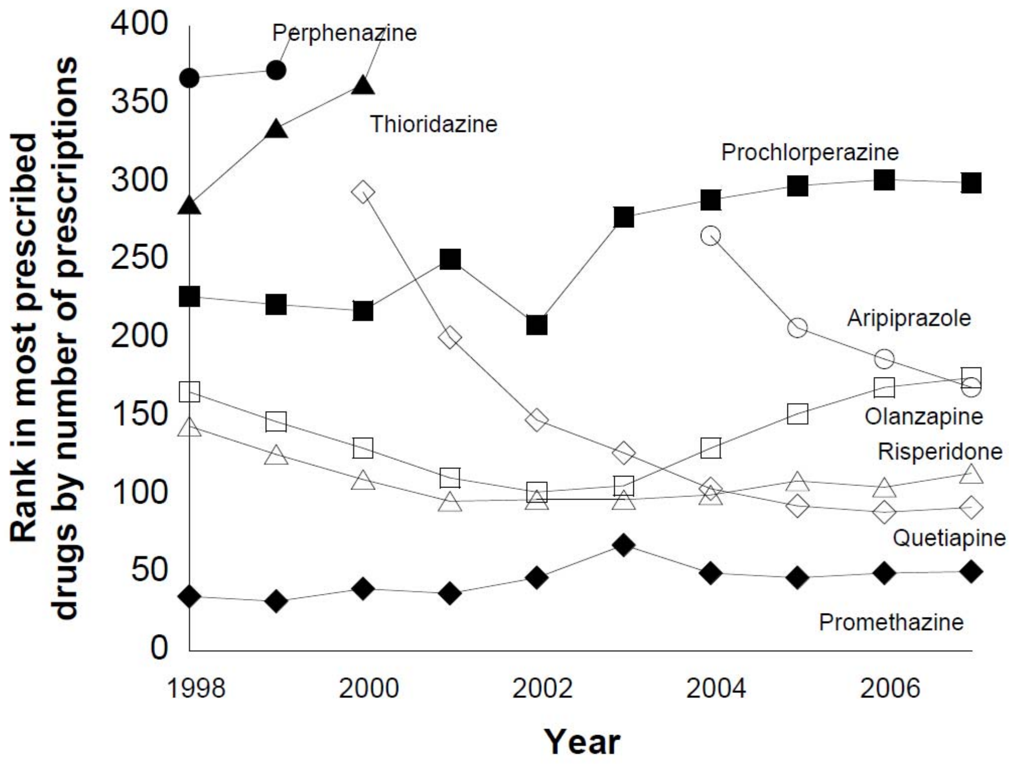

Figure S2-I. Rank of antipsychotic medications by total number of prescriptions in the United States in the time period from 1998-2007. Phenothiazines are indicated by closed symbols. Atypical antipsychotics are represented by open symbols. Prescriptions for atypical antipychotics over the last decade have been steadily increasing to the point that four of these drugs are among the top 200 most prescribed medications. There is only one phenothiazine left in the top 200 most prescribed medications (promethazine, formulated either alone or in combination with other drugs).
